# The Impact of the Antidiabetic Agent Sitagliptin on the Radiation Response of Breast Carcinoma Cells with Different Metastatic Capacities

**DOI:** 10.3390/cells15201879

**Published:** 2026-10-15

**Authors:** Marie Theres Eling, Anouk Bottema, Dragana Savic, Sergej Skvortsov, Ute Ganswindt, Petra Obexer, Ira Ida Skvortsova

**Affiliations:** 1 Department of Radiation Oncology, Medical University of Innsbruck, 6020 Innsbruck, Austria; anouk.bottema@i-med.ac.at (A.B.); dragana.savic@student.i-med.ac.at (D.S.); sergej.skvortsov@i-med.ac.at (S.S.); ute.ganswindt@i-med.ac.at (U.G.); ira.skvortsova@i-med.ac.at (I.I.S.); 2 Laboratory for Experimental and Translational Research on Radio-Oncology (EXTRO-Lab), Department of Radiation Oncology, Medical University of Innsbruck, 6020 Innsbruck, Austria; 3 Tyrolean Cancer Research Institute, 6020 Innsbruck, Austria; 4 Department of Pediatrics II, Medical University of Innsbruck, 6020 Innsbruck, Austria; petra.obexer@i-med.ac.at

**Keywords:** breast cancer cells, metastasis, radiotherapy, type 2 diabetes mellitus, DPP4/CD26, sitagliptin, cell survival, senescence, autophagy

## Abstract

Breast cancer (BC) and type 2 diabetes mellitus (T2DM) often co-occur and can contribute to poorer clinical outcomes after therapy compared to non-diabetic cases. It is assumed that glucose-lowering drugs like DPP4 inhibitors (DPP4-Is), e.g., sitagliptin, can affect the radiation responses of BC cells. In this study, parental and invasive (INV) MDA-MB-231 (triple-negative BC) and T47D (luminal A) cells were used. INV cells revealed a significant upregulation of DPP4/CD26 molecules compared to their parental counterparts. All investigated cells were treated with sitagliptin, ionizing radiation, or both. It was found that the specific inhibition of DPP4/CD26 molecules using sitagliptin can protect triple-negative BC cells from cytotoxic ionizing radiation or improve the radiation response in luminal A BC cells independently of their metastatic abilities. The protective and sensitizing effects of sitagliptin do not depend on the level of senescence and/or autophagy initiation after treatment.

## 1. Introduction

Breast cancer (BC) remains the second leading cause of cancer-related deaths among women in the European Union. According to estimates from the European Cancer Information System, this disease accounted for nearly 17% of all deaths of female patients from cancer in 2022, resulting in the loss of approximately 96,000 lives [1].

BC treatment has changed considerably over the last 20 years, largely due to progress in surgery, systemic therapies, and radiotherapy. Today, adjuvant radiation therapy (RT) is a standard part of care after breast-conserving surgery. Almost all patients receive RT, which can be given as either partial- or whole-breast irradiation, depending on patient and disease characteristics. This approach is now used routinely in daily clinical practice and has been shown to improve local disease control [2].

Type 2 diabetes mellitus (T2DM) represents a growing global health challenge, with prevalence rising at an alarming rate [3]. T2DM has gained increasing recognition as a factor influencing both the risk and prognosis of BC. Epidemiological studies indicate that T2DM is associated with a modestly increased risk of BC. However, this association is attenuated after adjusting for potential confounders such as age, body mass index, and geographic region. More robust evidence demonstrates that women with BC and pre-existing T2DM have significantly higher all-cause mortality compared to BC patients without diabetes [4,5,6].

Subtype-specific associations have also been reported, with T2DM linked to a higher likelihood of developing triple-negative breast cancer (TNBC) [7]. This observation is consistent with preclinical data suggesting a stronger biological interplay between diabetes and TNBC pathogenesis. Furthermore, the reduced efficacy of anticancer treatments in BC patients with concurrent T2DM has emerged as a critical determinant of their less favorable clinical outcomes, which is increasingly drawing attention in cancer research [8,9,10,11].

Over the past decade, dipeptidyl peptidase-4 inhibitors (DPP-4is, also known as “gliptins”) have become established oral agents for the management of T2DM. These drugs act by blocking the enzymatic activity of DPP-4, thereby prolonging the half-life of incretin hormones, such as glucagon-like peptide-1 (GLP-1) and glucose-dependent insulinotropic peptide (GIP). Through this mechanism, they help restore the key metabolic functions impaired in diabetes. Sitagliptin was the first substance of this class approved by the U.S. Food and Drug Administration (FDA), either as monotherapy or in combination with metformin [3].

DPP-4 (also referred to as CD26) is a cell surface aminopeptidase expressed in both transmembrane and soluble forms across multiple cell types [12]. The membrane-bound form cleaves incretins, chemokines, and inflammatory mediators, while the soluble form has been linked to inflammation, obesity, insulin resistance, and metabolic syndrome [13]. Beyond metabolism, DPP-4 is highly expressed in several malignancies, including BC, and plays a critical role in immune regulation through its expression on T, B, and natural killer (NK) cells and macrophages [14]. Altered CD26 expression on lymphocytes has been associated with BC incidence, thus suggesting an immunological connection between DPP-4 and tumor biology [15].

Overall, current evidence highlights the dual role of DPP-4 in cancer as both a regulator of immune cell infiltration into the tumor microenvironment and a modulator of metastasis through CXCL12 cleavage [16]. However, the clinical significance of DPP-4 inhibition in patients with concurrent diabetes and cancer remains unresolved. This raises important concerns regarding the oncological safety of DPP-4is and underscores the need for further studies.

While the inhibition of DPP-4 is a well-established therapeutic strategy for T2DM, its impact on tumor biology remains complex and controversial [15,17,18,19]. Contrary to the initial expectation that DPP-4 blockade might suppress tumor-promoting functions, emerging evidence suggests that DPP-4is may instead facilitate cancer progression [15]. Preclinical studies have demonstrated that DPP-4is can promote BC metastasis, invasion, epithelial–mesenchymal transition (EMT), and chemoresistance, primarily through the activation of the CXCL12/CXCR4/mTOR signaling axis [17]. In line with these findings, Kawakita et al. (2023) reported that DPP-4is enhance BC cell survival via the induction of autophagy [18]. Clinically, treatment with DPP-4is has been associated with poorer survival outcomes in BC patients, although the findings remain inconsistent across studies [15,16,19,20]. Given that DPP-4 is often upregulated in carcinoma cells, with particular importance in the context of metastases, we hypothesized that invasive (INV) BC cell models [21] would also display enhanced DPP-4 expression. Such an assumption is consistent with the enzyme’s reported involvement in tumor-associated processes, including proliferation, migration, and invasion [16]. Investigating the expression of DPP-4 in these models may therefore provide valuable insights into its role in BC aggressiveness. Based on our earlier findings demonstrating that INV breast cancer cells not only possess a higher metastatic potential but also display increased resistance to ionizing radiation compared with their less aggressive parental counterparts [21], we employed INV cells to assess the radiation response of BC cells in the presence of the DPP4i sitagliptin. In this study, we investigated the functional role of sitagliptin and its impact on the efficacy of ionizing irradiation.

## 2. Materials and Methods

### 2.1. Cell Culture

MDA-MB-231 (triple-negative type (TNBC): estrogen, progesterone, and HER2/neu receptor-negative (ER−, PR−, and HER2/neu−)) and T47D (luminal A type: ER+, PR+, and HER2/neu−) BC cells were purchased from the American Type Culture Collection. All cell lines were grown in RPMI1640 medium (Thermo Fisher Scientific, Waltham, MA, USA) supplemented with 2 mM of L-glutamine, 50 U/mL of penicillin, 50 μg/mL of streptomycin (penicillin–streptomycin–glutamine (100×); Thermo Fisher Scientific, Waltham, MA, USA), and 10% fetal bovine serum (HyClone Standard FBS, US Origin, 500 mL, irradiated at 25–40 kGy, heat-inactivated; Cytiva, Marlborough, MA, USA). To culture T47D cells, insulin solution (from the bovine pancreas; Sigma-Aldrich, St. Louis, MO, USA) was additionally added to the media. Cell cultures were maintained in a 5% CO_2_ humidified atmosphere at 37 °C. BC cells with increased invasive potential (INV cells) were generated from the parental BC cell lines following the procedure previously reported by our group [21]. The INV cells were maintained under standard culture conditions as described above.

### 2.2. Cell Treatment with Sitagliptin and Ionizing Irradiation

Sitagliptin in the form of phosphate monohydrate, which is an active ingredient in the antidiabetic drug Januvia, was used at a clinically relevant dose of 4 µM. This dose was calculated based on the daily prescription of medication (100–200 mg of Januvia per day). It also corresponds to the data described in a recent report by Herman, G.A. et al. (2011) on 1–5 µM plasma sitagliptin concentrations following single oral doses of 200–400 mg after 12–24 h [22,23,24,25]. Sitagliptin was applied 24 h prior to irradiation to give the cells time to successfully inhibit DPPIV activities. Furthermore, this better simulates the clinical situation of breast cancer patients continuously receiving sitagliptin when starting radiotherapy. The medium containing 4 µM of sitagliptin was not changed for 72 h in the viability assay, but it was changed twice in the clonogenic survival assay.

On day 0, the cells were seeded in appropriate plates. On the next day, the medium was changed using culture media supplemented with 4 µM of sitagliptin (Sitagliptin Phosphate Monohydrate; Abcam, Cambridge, UK; diluted in DMSO) or DMSO (Sigma-Aldrich, St. Louis, MO, USA; 0.01%) as a vehicle control. On day 2, the cells were irradiated at single doses of 2 or 8 Gy (16 MV X-rays) using an Elekta Precise Linear Accelerator (Elekta Oncology Systems, Crawley, UK) at a dose rate of approximately 1.8 Gy/min with a Source-to-Surface Distance (SSD) of 100 cm and Field of View (FOV) of 40 × 40 cm. A 1 cm solid water phantom on top of the plates was used to ensure accurate dose delivery. Cells were exposed to irradiation at room temperature, but no irradiation was used for control samples. After irradiation, the cells were immediately placed in an incubator for further culturing.

### 2.3. Flow Cytometry

To analyze DPP4/CD26 expression in parental and invasive MDA-MB-231 and T47D cells using flow cytometry (MACSQuant^®^ 16 Analyzer; Miltenyi Biotec, Bergisch-Gladbach, Germany), 1 × 10^6^ cells were pelleted at 500× *g*, resuspended, and stained with 2 µL of CD26 antibody (anti-human, PE; Miltenyi Biotec, Bergisch-Gladbach, Germany). An equivalent amount of PE-conjugated IgG2a antibody (Isotype Control Antibody, mouse IgG2a, PE; Miltenyi Biotec, Bergisch-Gladbach, Germany) was used as the isotype control. Labeled cells were washed in FACS buffer and then analyzed using flow cytometry, and the results were subsequently evaluated via FlowJo™ Software v11. All measurements were repeated at least three times. The gating strategy can be found in the Supplemental Materials.

### 2.4. Cell Viability Assay

To evaluate cell viability, BC cells were seeded in 6–well plates (100,000 cells/well) and allowed to recover and adhere overnight. On the next day, the medium was changed using culture media supplemented with 4 µM of sitagliptin (Sitagliptin Phosphate Monohydrate; Abcam, Cambridge, UK; diluted in DMSO) or DMSO (Sigma-Aldrich, St. Louis, MO, USA; 0.01%) as a vehicle control. On day 2, the cells were irradiated at single doses of 2 or 8 Gy as mentioned above. After the treatment period of 72 h, cell viability was evaluated using a test based on trypan blue dye exclusion as previously described [26,27].

Briefly, cells were trypsinized and counted using a Beckman Coulter Vi-CELL AS cell viability analyzer (Beckman Coulter, Fullerton, CA, USA). The number of viable cells was determined in the control and treated BC cell samples. Cell viability was represented as the percentage of viable cells relative to the untreated control cells.

### 2.5. Clonogenic Cell Survival Assay

To investigate the ability of the cells to form colonies after treatment, both parental and invasive BC cells were seeded in 6-well plates. On the next day, the medium was changed using culture media supplemented with 4 µM of sitagliptin (Sitagliptin Phosphate Monohydrate; Abcam, Cambridge, UK; diluted in DMSO) or DMSO (Sigma-Aldrich, St. Louis, MO, USA; 0.01%) as a vehicle control. On day 2, the cells were irradiated at single doses of 2 or 8 Gy as mentioned above. After 7 (MDA-MB-231) or 14 (T47D) days, the colonies were stained with 1% crystal violet in 20% methanol, and those with over 50 cells were counted with a ColCount colony counter (Oxford Optronix, Oxford, UK). Individual assays were performed in duplicate and repeated at least three times. The surviving fraction (SF) was estimated using the following formula:

Plating efficiency (PE) of the untreated control was calculated as follows:

PE = number of colonies formed/number of cells seeded

The surviving fraction (SF) of the treated cells was calculated by normalizing colony formation to the PE of the corresponding untreated control:

SF = number of colonies formed/(number of cells seeded × PE of the untreated control)

Treatment–response clonogenic survival curves were plotted using a linear–quadratic model [28].

### 2.6. RT-qPCR Analysis

Cells were seeded in 6-well plates. On the next day, the medium was changed using culture media supplemented with 4 µM of sitagliptin (Sitagliptin Phosphate Monohydrate; Abcam, Cambridge, UK; diluted in DMSO) or DMSO (Sigma-Aldrich, St. Louis, MO, USA; 0.01%) as a vehicle control. On day 2, the cells were irradiated at single doses of 2 or 8 Gy as mentioned above. After 72 h, the cells were detached using 300 µL of trypsin and washed twice with cold PBS in 1.5 mL tubes (AXYGEN^®^; Avantor, Radnor, PA, USA). The cells were centrifuged at 4 °C at 2000 rpm for 10 min, and the pellets were snap-frozen in liquid nitrogen and stored at −80 °C until further use. RNA was isolated from the cell pellets using the RNeasy Plus Micro Kit (Qiagen, Hilden, Germany) according to the manufacturer’s protocol. RNA concentrations were measured using Nanodrop (Nanodrop 1000 Spectrophotometer, ND-1000; Peqlab, Erlangen, Germany).

Afterwards, 1 µg of first-strand cDNA per sample was synthesized using SuperScript 2 Reverse Transcriptase (Invitrogen, Thermo Fisher Scientific, Waltham, MA, USA) according to the manufacturer’s protocol. Primers for senescence (Hs_GLB1_1_SG QuantiTect Primer Assay; GeneGlobe ID—QT00066206; Qiagen, Hilden, Germany), autophagy (Hs_MAP1LC3A_1_SG QuantiTect Primer Assay; GeneGlobe ID—QT00234017; Qiagen, Hilden, Germany), and the housekeeping gene importin 8 (Hs_IPO8_1_SG QuantiTect Primer Assay; GeneGlobe ID—QT00071246; Qiagen, Hilden, Germany) were used together with SensiFAST Sybr Fluorescein Kit (Labconsulting, Vienna, Austria) and measured with 2 ng/µL of cDNA in the qPCR (iQ™5 Real-Time PCR Detection Systems; BioRad, Hercules, CA, USA). The results were calculated using the 2^−ΔΔCt^ method and normalized to importin (IPO8), as well as the untreated control.

### 2.7. Senescence Activity Assay

For this experiment, the Mammalian β-Galactosidase Assay Kit (Thermo Fisher Scientific, Waltham, MA, USA) was used. BC cells were seeded in 96-well plates. On the next day, the medium was changed using culture media supplemented with 4 µM of sitagliptin (Sitagliptin Phosphate Monohydrate; Abcam, Cambridge, UK; diluted in DMSO) or DMSO (Sigma-Aldrich, St. Louis, MO, USA; 0.01%) as a vehicle control. On day 2, the cells were irradiated at single doses of 2 or 8 as mentioned above. After 72 h, the cells were washed and lysed according to the manufacturer’s protocol. Subsequently, the investigated cells were incubated with 50 µL of β-Gal Assay Reagent for 30 min at 37 °C. After incubation, absorbance was measured at 405 nm using a microplate reader (Synergy H1 Plate Reader, BioTek, Atlanta, GA, USA).

### 2.8. Evaluation of SASP-Associated Protein Levels

The Senescence-Associated Secretory Phenotype (SASP) ELISA Sampler Kit (Assay Genie, Dublin, Ireland) was used to determine the expression of the SASP-associated proteins IL-1-beta, IL-6, MMP3, PAI1, CXCL10/IP10, MCP-1/CCL2, and TNF alpha. BC cells were seeded in 6-well plates. On the next day, the medium was changed using culture media supplemented with 4 µM of sitagliptin (Sitagliptin Phosphate Monohydrate; Abcam, Cambridge, UK; diluted in DMSO) or DMSO (Sigma-Aldrich, St. Louis, MO, USA; 0.01%) as a vehicle control. On day 2, the cells were irradiated at single doses of 2 or 8 Gy as mentioned above. Afterwards, the cells were lysed at 72 h. This experiment was performed according to the manufacturer’s protocol. Absorbance was measured at 450 nm using a microplate reader (Synergy H1 Plate Reader, BioTek, Atlanta, GA, USA). The results were normalized to the protein concentration, and protein expression levels were calculated according to the formula from the standard curve measured with each sample run.

### 2.9. Autophagy Activity Assay

Autophagy Assay Kit (Abcam, Cambridge, UK) was used to determine whether BC cells reveal autophagy development after treatment with sitagliptin or irradiation alone or both. Measurements were performed following the manufacturer’s protocol. Briefly, the reagents were prepared as suggested in the instructions. Cells were seeded (5000 or 10,000 cells/well) in 96-well plates. On the next day, the medium was changed using culture media supplemented with 4 µM of sitagliptin (Sitagliptin Phosphate Monohydrate; Abcam, Cambridge, UK; diluted in DMSO) or DMSO (Sigma-Aldrich, St. Louis, MO, USA; 0.01%) as a vehicle control. On day 2, the cells were irradiated at single doses of 2 or 8 Gy as mentioned above. Autophagy was measured 72 h after treatment. Untreated and treated cells were washed with assay buffer and suspended in a dual-color detection solution for 30 min at 37 °C in the absence of light. Cells were washed twice with 200 µL of assay buffer, 100 µL of assay buffer was added, and fluorescence levels were then analyzed using the microplate reader (Hidex Sense Microplate Reader, Hidex, Turku, Finland). Autophagosomes were stained using green detection reagent (FITC filter: excitation 480 nm, emission 530 nm), and nuclei were detected via nuclear staining (DAPI filter: excitation 340 nm, emission 480 nm). The fold change in autophagy development was calculated as the ratio between the signal in the treated and untreated samples.

Next, the Autophagy Assay Red Detection Kit (Bio-Rad, Hercules, CA, USA) was used. BC cells were seeded in 6-well plates. On the next day, the medium was changed using culture media supplemented with 4 µM of sitagliptin (Sitagliptin Phosphate Monohydrate; Abcam, Cambridge, UK; diluted in DMSO) or DMSO (Sigma-Aldrich, St. Louis, MO, USA; 0.01%) as a vehicle control. On day 2, the cells were irradiated at single doses of 2 or 8 Gy as mentioned above. After 72 h, the cells were washed with fresh culture media and stained with Autophagy Probe Red at a ratio of 1:50. After 1 h of incubation at 37 °C without light exposure, the cells were trypsinized, washed, and analyzed using flow cytometry (MACSQuant^®^ 16 Analyzer; Miltenyi Biotec, Bergisch-Gladbach, Germany) according to the manufacturer’s protocol. As a negative control, 0.01% DMSO (Sigma-Aldrich, St. Louis, MO, USA) was used, and as a positive control, the cells were treated with Rapamycin (500 nM) and Chloroquine (60 µM) for 24 h. The gating strategy can be found in the Supplemental Material. The results were subsequently evaluated using FlowJo™ Software v11. All measurements were repeated at least twice with technical duplicates.

### 2.10. Statistical Analysis

Graphs were made and statistical analyses were carried out using GraphPad Prism (version 10). Data are expressed as the mean  ±  standard deviation (SD). Statistical analysis was performed using factorial analysis of variance (ANOVA). For experiments assessing the effects of cell line, sitagliptin treatment (0 or 4 µM), and irradiation dose (0, 2, or 8 Gy), a three-way ANOVA was applied, including all main effects and two- and three-way interactions. For experiments involving only two experimental factors, a two-way ANOVA including both main effects and their interaction was used. Biological replicates were considered independent experimental units; where technical replicates were performed, these were averaged before statistical analysis and were not treated as independent observations. Following ANOVA, model-based pairwise post-hoc comparisons were performed where appropriate, and *p* values were adjusted for multiple testing using the Holm step-down procedure. All tests were two-sided, and adjusted *p* < 0.05 was considered statistically significant: * *p*  ≤  0.05, ** *p*  ≤  0.01, *** *p*  ≤  0.001.

## 3. Results

### 3.1. CD26/DPP4 Expression in BC Cells

**Figure 1:**
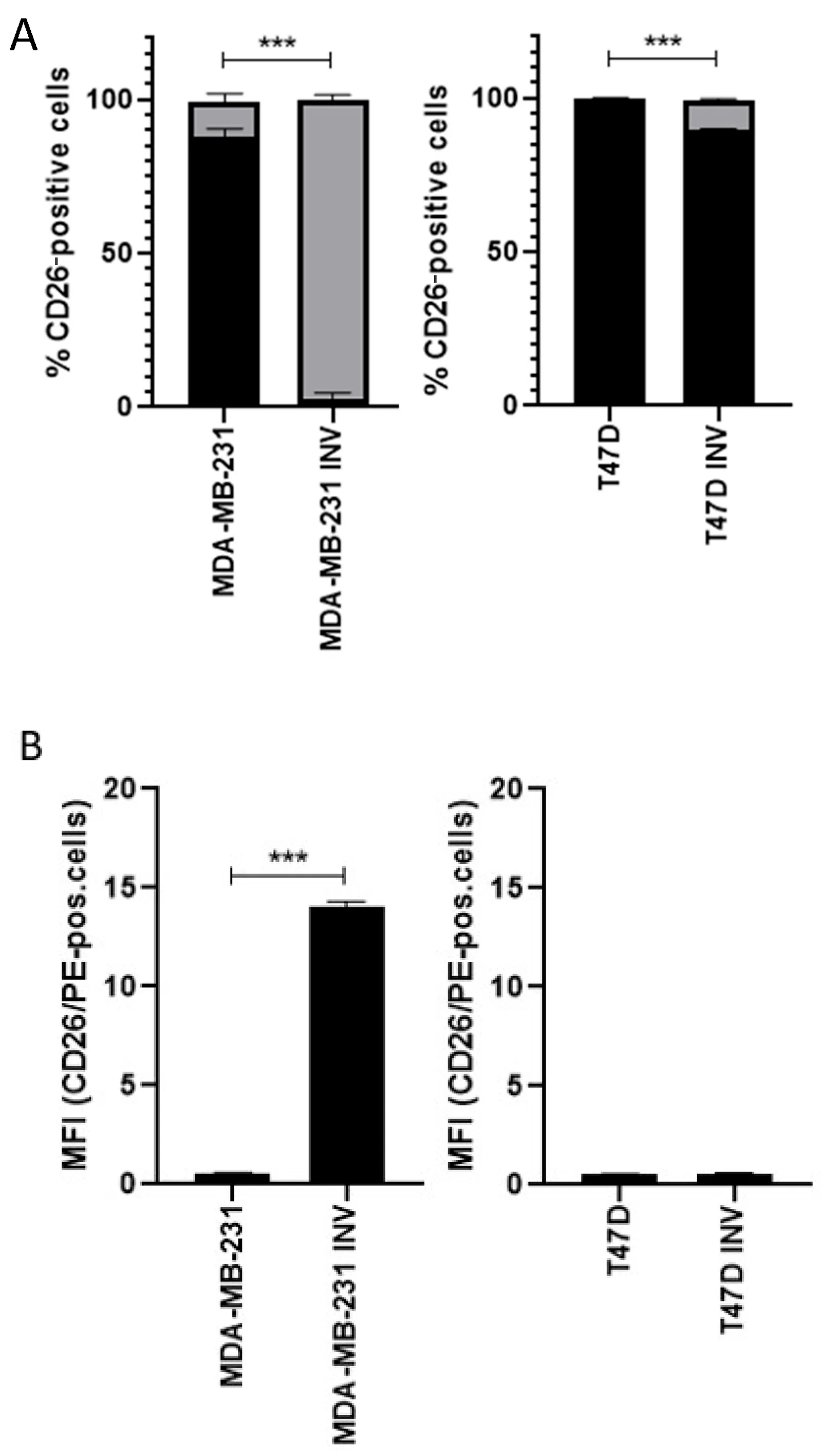
DPP4/CD26 expression in BC cells. Columns indicate mean values presented as mean ± standard deviation from three independent experiments containing two technical replicates (*n* = 6) (*** *p* < 0.001). (**A**) Black and gray bars represent CD26-negative and -positive cells in %, respectively. (**B**) Black bars represent the mean fluorescence intensity (MFI) from CD26/PE-positive cells.

To determine whether BC cells with enhanced invasive capacities possess any differences in DPP4/CD26 expression in comparison with parental cells, FACS analysis was performed. The percentage of CD26-positive cells is presented in Figure 1A, while the corresponding mean fluorescence intensity (MFI) is shown in Figure 1B as a measure of CD26 expression on BC cells. As shown in Figure 1, MDA-MB-231 INV and T47D INV cells exhibited significantly higher percentages of CD26-positive cells compared to their parental counterparts. Thus, MDA-MB-231-INV cells contained 96.85 ± 1.85% CD26-positive cells vs. 11.45 ± 2.95% in parental MDA-MB-231 cells. This difference was further supported by a significantly increased MFI in MDA-MB-231-INV cells compared to their parental counterparts (Figure 1B). Two-way ANOVA confirmed a significant cell line × CD26 status interaction (*p* = 2.63 × 10^−6^), and Holm-adjusted post hoc comparisons demonstrated significant differences between parental and INV cells for both the CD26-positive and CD26-negative populations (both *p* = 2.52 × 10^−6^). T47D cells also demonstrated an upregulation of CD26 expression, with 1.18 ± 0.52% and 9.96 ± 0.44% CD26-positive cells in parental and invasive T47D BC cells, respectively. In contrast to the MDA-MB-231 model, no significant difference in MFI was observed in invasive T47D cells in comparison with parental T47D cells (Figure 1B). Two-way ANOVA revealed a significant cell line × CD26 status interaction (*p* = 7.59 × 10^−6^), and Holm-adjusted post-hoc comparisons confirmed significant differences between parental and INV cells for both the CD26-positive (*p* = 1.68 × 10^−5^) and CD26-negative populations (*p* = 5.29 × 10^−5^).

### 3.2. Differences in Survival of BC Cells Treated with Sitagliptin and Ionizing Irradiation

**Figure 2:**
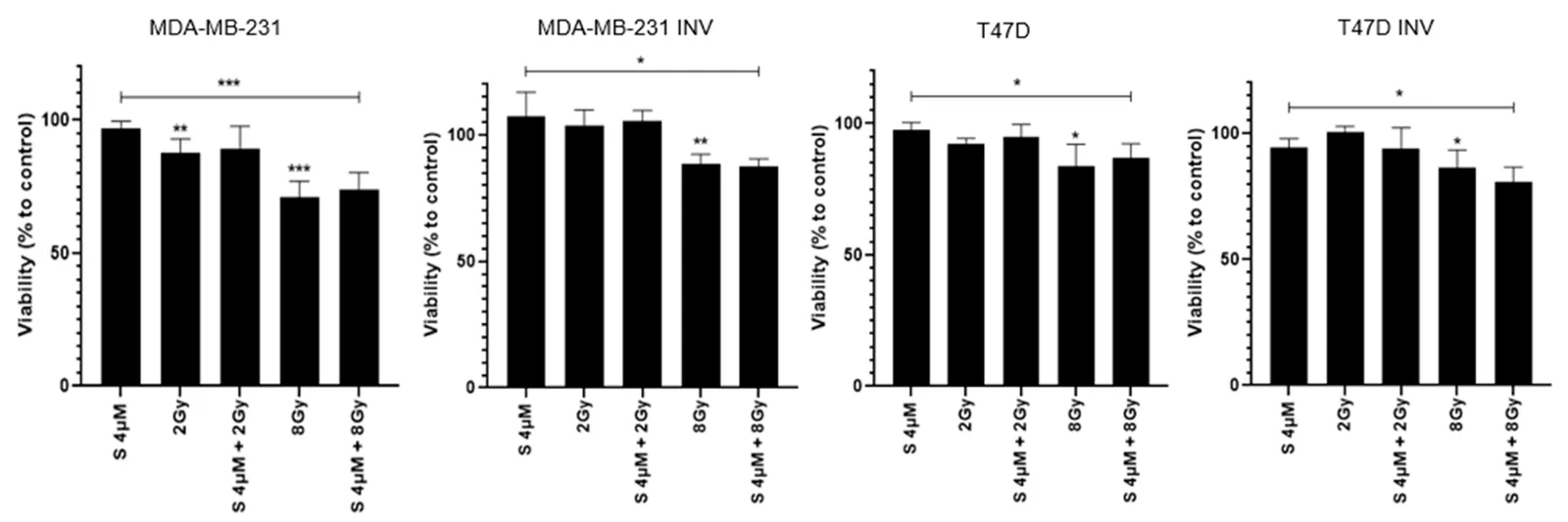
Influence of sitagliptin, irradiation, or both on viability of BC cells. Parental and INV BC cells were treated with 4 µM sitagliptin alone, irradiation at single doses of 2 or 8 Gy, or both. Cell viabilities were evaluated at 72 h after treatment. Columns indicate cell viability (%) relative to untreated control, which is presented as mean ± standard deviation from four independent experiments containing two technical replicates (*n* = 8). Statistical significance indicated directly above individual bars represents comparisons with the corresponding untreated control, whereas significance indicated above horizontal brackets represents comparisons between the treatment groups connected by the respective bracket. (* *p* < 0.05; ** *p* < 0.01; *** *p* < 0.001).

Short-term cell viabilities and long-term reproductive (clonogenic) cell survival rates were assessed to determine the treatment responses of the investigated BC cells. As illustrated in Figure 2, no BC cells showed any significant changes in short-term viability after treatment with sitagliptin (4 µM) alone.

Following ionizing irradiation at 2 Gy, both parental cells showed no significantly reduced cell viabilities. Cell exposure to a higher dose of irradiation (8 Gy) resulted in decreased cell viabilities in parental MDA-MB-231 and T47D cells at 72 h after treatment (70.94% ± 3.73% and 83.79% ± 9.06%, respectively). The cell viability of neither MDA-MB-231-INV nor T47D-INV cells was affected after their exposure to irradiation at a single dose of 2 Gy compared to non-irradiated cells. However, INV cell irradiation at a dose of 8 Gy resulted in a decrease in cell viabilities (88.29% ± 3.19% for MDA-MB-231-INV and 86.27% ± 10.45% for T47D-INV). In the MDA-MB-231 model, ANOVA confirmed significant effects of radiation dose and cell line, as well as a significant cell line × radiation dose interaction. Holm-adjusted post-hoc comparisons showed significantly lower viability in parental MDA-MB-231 cells compared to MDA-MB-231-INV cells following 2 Gy (*p* = 0.00138) and 8 Gy (*p* = 0.000577), as well as following sitagliptin combined with 2 Gy (*p* = 0.00132) and 8 Gy (*p* = 0.00913). No significant overall effect of sitagliptin or sitagliptin × radiation dose interaction was detected. In the T47D model, ANOVA confirmed a significant effect of radiation dose and identified a cell line × sitagliptin interaction, while no significant overall effects of cell line or sitagliptin and no sitagliptin × radiation dose interaction were observed. Consistently, Holm-adjusted comparisons revealed no significant differences between parental T47D and T47D-INV cells under matched treatment conditions. When sitagliptin was combined with irradiation at a dose of 2 Gy, it yielded a similar trend; however, these differences did not reach statistical significance compared to the irradiated-only cells.

In contrast, combination treatment using sitagliptin and irradiation at 8 Gy led to significantly lower cell viabilities compared to sitagliptin alone (~1.3-fold for both parental MDA-MB-231 and MDA-MB-231-INV cells and ~1.3-fold for the parental T47D cells and ~1.2-fold for T47D-INV cells).

**Figure 3:**
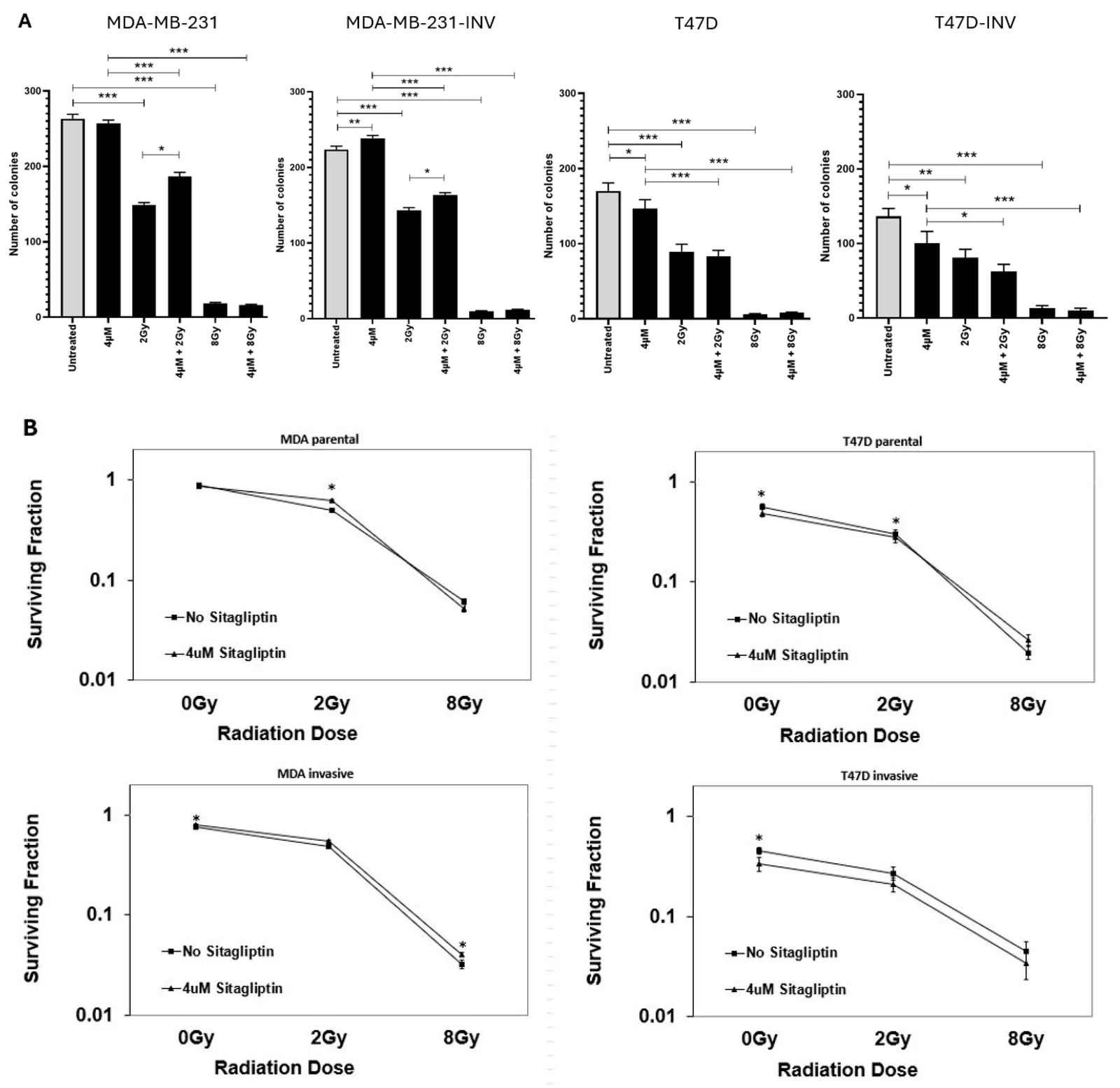
The clonogenic survival of BC cells after treatment with sitagliptin, ionizing radiation, or both. (**A**) The bars show the number of colonies: gray represents the untreated control and black represents the treated cells. The bars are presented as the mean ± standard deviation after three independent experiments containing three technical replicates (*n* = 9). (**B**) The surviving fractions were plotted as a function of irradiation dose on a linear–quadratic model. The error bars indicate the standard deviations of the means obtained after technical triplicate measurements from three independent experiments (*n* = 9) (* *p* < 0.05; ** *p* < 0.01; *** *p* < 0.001).

Long-term reproductive clonogenic survival was evaluated in parental and INV BC cells after their exposure to sitagliptin, ionizing irradiation, or both (Figure 3A,B). Parental and INV breast carcinoma cells showed comparable responses to irradiation at doses of 2 Gy and 8 Gy, with decreased clonogenic survival after cell exposure to higher doses of irradiation. In parental MDA-MB-231 cells, the surviving fraction decreased from 0.878 ± 0.116 in non-irradiated cells to 0.497 ± 0.089 after irradiation at 2 Gy (Holm-adjusted *p* = 4.60 × 10^−7^) and 0.061 ± 0.040 following 8 Gy (*p* = 1.36 × 10^−13^). A comparable radiation-dependent reduction was observed in MDA-MB-231-INV cells. ANOVA confirmed significant effects of radiation dose and cell line, whereas no significant main effect of sitagliptin or interaction between sitagliptin and radiation dose was detected. Despite these comparable radiation responses, both cell types showed distinct patterns following sitagliptin treatment. In parental MDA-MB-231 cells, clonogenic capacity remained unchanged upon sitagliptin exposure. Parental T47D cells showed a slightly lower clonogenic capacity upon treatment with sitagliptin. In parental T47D cells, the surviving fraction decreased from 0.568 ± 0.092 in non-irradiated cells to 0.298 ± 0.118 after cell exposure to 2 Gy (Holm-adjusted *p* = 0.0090) and 0.0196 ± 0.0089 after irradiation at 8 Gy (*p* = 2.40 × 10^−7^). ANOVA confirmed significant effects of radiation dose and cell line, but no significant main effect of sitagliptin or interaction between sitagliptin and radiation dose.

In line with this, subtype-specific effects were observed in INV cells. Triple-negative MDA-MB-231-INV cells demonstrated enhanced clonogenic survival after sitagliptin treatment compared to their parental counterparts. In contrast, sitagliptin caused a ~1.4-fold reduction in clone formation in luminal A T47D-INV cells compared to the untreated control.

Combination treatment using sitagliptin and irradiation at a single dose of 2 Gy resulted in an enhancement in clonogenic survival in both parental and invasive MDA-MB-231 cells compared to irradiation alone (~1.3- and ~1.2-fold, respectively). Clonogenic survival in cells treated with sitagliptin combined with ionizing radiation at a dose of 8 Gy did not significantly differ from that in cells treated with irradiation alone.

Parental and invasive T47D cells were not affected in terms of their ability to form clones after treatment with the combination of sitagliptin and irradiation at a single dose of 2 Gy compared to irradiation alone, but significantly fewer clones were formed in comparison with sitagliptin alone. The combination treatment of sitagliptin and irradiation at a single dose of 8 Gy led to slightly but significantly better clonogenic survival in parental T47D cells compared to irradiation alone. T47D-INV cells did not show significant differences after combination treatment compared to irradiation alone. Holm-adjusted comparisons revealed no significant differences between parental and INV cells under matched treatment conditions.

### 3.3. Sitagliptin, Ionizing Radiation, and Senescence Initiation

**Figure 4:**
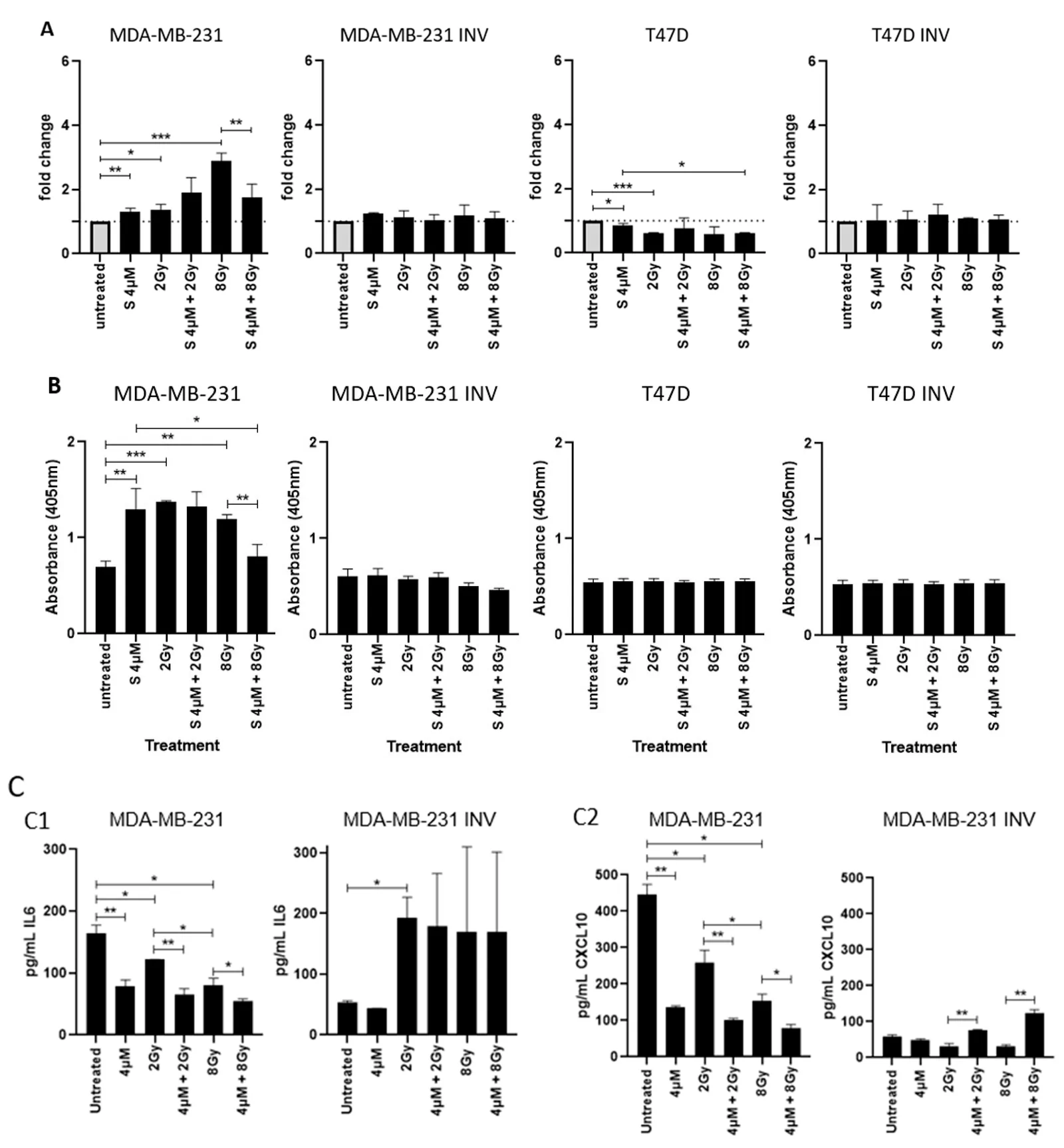

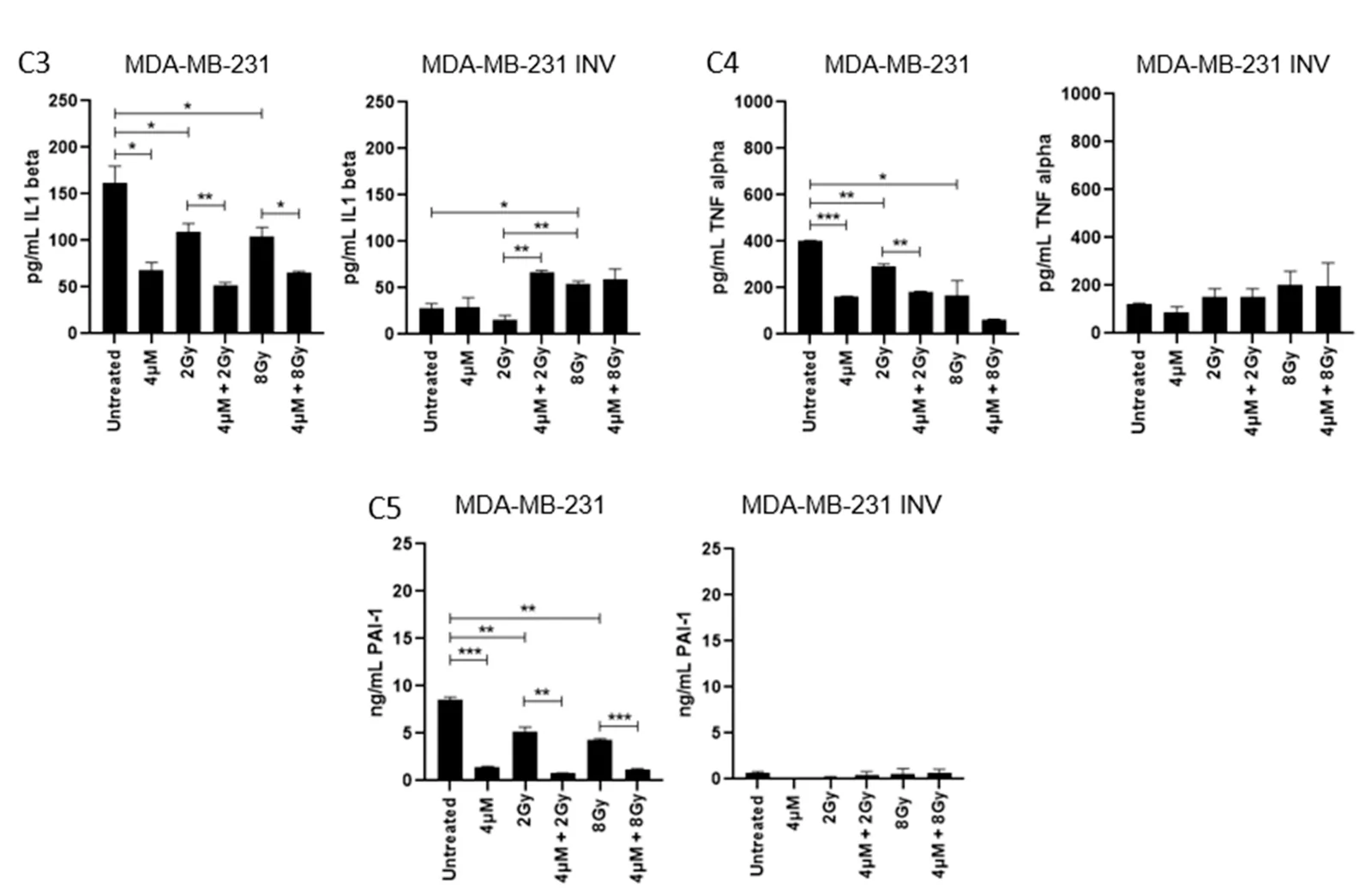
Senescence development in BC cells after treatment with sitagliptin, irradiation, or both. (**A**) Glb1 gene expression was detected using the iQ™5 Real-Time PCR Detection System, 72 h after treatment with sitagliptin (4 µM) or irradiation (2 or 8 Gy) alone or both. ΔΔCt-values were also calculated. The bars are presented as the mean ± standard deviation of the fold change vs. the untreated control normalized to the housekeeping gene importin 8 (IPO8) after three independent experiments containing two technical replicates (*n* = 6). The gray bars show the untreated control, and the black bars represent the treated cells. A dashed line at a fold change of 1 indicates the level of the untreated control to facilitate visualization of differences relative to the untreated control. (**B**) The expression of the GLB1 protein in BC cells treated as described in Section 2.7 of the Materials and Methods. Columns represent the mean absorbance values. The bars are presented as the mean ± standard deviation after three independent experiments containing two technical replicates (*n* = 6). (**C**) The expression of the SASP proteins IL6 (**C1**), CXCL10/IP10 (**C2**), IL1 beta (**C3**), TNF alpha (**C4**), and PAI-1 (**C5**) in BC cells treated as described in Section 2.8 of the Materials and Methods. Columns represent the mean absorbance values normalized to the protein concentration and calculated according to the formula of the standard curve. The y-axis shows the range of each protein from low to high expression according to the standard measurement. The bars are presented as the mean ± standard deviation from two independent experiments (*n* = 2) (* *p* < 0.05; ** *p* < 0.01; *** *p* < 0.001).

Subsequently, we investigated whether BC cells undergo senescence following the administration of sitagliptin, ionizing radiation, or both (Figure 4).

The analysis of galactosidase beta 1 (GLB1) gene expression (Figure 4A) following sitagliptin treatment alone revealed a slight, but significant, upregulation in parental MDA-MB-231 cells (1.31 ± 0.14-fold change) compared to the untreated control. In contrast, parental T47D cells showed a downregulation of the GLB1 gene (0.86 ± 0.04-fold change) compared to the untreated control. Neither MDA-MB-231-INV nor T47D-INV cells demonstrated any differences in GLB1 gene expression levels compared to their corresponding untreated controls.

A significant dose-dependent upregulation of the GLB1 gene was observed in parental MDA-MB-231 cells upon irradiation alone (1.37 ± 0.12-fold change for 2 Gy and 2.89 ± 0.22-fold change for 8 Gy) compared to the untreated control. MDA-MB-231-INV cells with enhanced metastatic capacities were not altered in terms of their GLB1 gene expression after irradiation at both doses compared to untreated control cells. Consistent with these differential responses, three-way ANOVA revealed significant effects of cell line and radiation dose, as well as significant cell line × radiation dose, sitagliptin × radiation dose, and three-way interactions in the MDA-MB-231 model.

In contrast, parental T47D cells exhibited a significant downregulation of GLB1 gene expression in response to irradiation at 2 Gy (0.61 ± 0.02-fold change) compared to the untreated control, whereas cell exposure to irradiation at 8 Gy did not significantly change GLB1 gene expression. T47D-INV cells did not demonstrate any differences in their GLB1 expression levels after irradiation at any dose. In the T47D model, three-way ANOVA identified a significant overall effect of cell line, but no significant effects of sitagliptin or radiation dose and no significant interactions. Accordingly, Holm-adjusted post-hoc analysis revealed no significant differences between parental T47D and T47D-INV cells under matched treatment conditions.

Combination treatment with sitagliptin and irradiation at a dose of 2 Gy did not induce significant alterations in GLB1 expression in all investigated cells compared to irradiation or sitagliptin used alone. However, the combination of sitagliptin and a higher dose of irradiation (8 Gy) led to the downregulation of GLB1 gene expression in parental MDA-MB-231 cells compared to irradiation alone. Specifically, the addition of sitagliptin significantly reduced GLB1 expression in parental MDA-MB-231 cells after their exposure to irradiation at 8 Gy from 2.898 ± 0.241-fold to 1.764 ± 0.404-fold (Holm-adjusted *p* = 0.000408). In contrast, the addition of sitagliptin to irradiation did not significantly alter GLB1 expression in both parental and invasive T47D cell lines after Holm correction. Both invasive cell lines, MDA-MB-231-INV and T47D-INV, displayed no differences in GLB1 gene expression after their exposure to any kind of treatment.

To understand whether the treatment-produced dysregulations in GLB1 gene expression translated to any alterations in GLB1 activities, a GLB1 assay was used (Figure 4B). It was found that among the investigated cells, only the parental MDA-MB-231 cells demonstrated an upregulation of GLB1 activity after treatment. Sitagliptin alone increased GLB1 activity by 1.86 ± 0.17-fold compared to the untreated control. Both doses of irradiation also amplified GLB1 activity by 1.99 ± 0.20-fold for 2 Gy and 1.73 ± 0.17-fold for 8 Gy. ANOVA confirmed significant effects of cell line and radiation dose, as well as significant cell line × radiation dose, sitagliptin × radiation dose, and three-way interactions. Although the combination of sitagliptin and ionizing radiation at a dose of 2 Gy did not markedly change GLB1 activity compared to sitagliptin or irradiation alone, the use of this drug with a higher dose of ionizing radiation at 8 Gy led to a reduction in GLB1 activity compared to sitagliptin or irradiation alone (~1.5- and ~1.3-fold, respectively). No significant treatment-associated differences in GLB1 activity were detected in MDA-MB-231-INV, T47D, or T47D-INV cells after Holm correction. Consistently, no significant main effects or interactions were detected in the T47D model.

To further investigate the induction of senescence in BC cells following the different treatments, SASP proteins were analyzed. The corresponding results are presented in Figure 4C.

Untreated parental MDA-MB-231 cells exhibited the constitutive expression of IL6 (164.9 ± 12.8 pg/mL), CXCL10/IP10 (446.3 ± 27.8 pg/mL), IL1β (162.4 ± 17.5 pg/mL), TNFα (401.9 ± 2.6 pg/mL), and PAI-1 (8.6 ± 0.3 ng/mL), whereas MCP-1/CCL2 and MMP3 were not detected.

Treatment with sitagliptin alone in parental MDA-MB-231 cells resulted in a downregulation of IL6 (79.6 ± 10 pg/mL), CXCL10/IP10 (136.9 ± 3.4 pg/mL), IL1β (68.1 ± 8.3 pg/mL), TNFα (163.3 ± 1.5 pg/mL), and PAI-1 (1.5 ± 0.1 ng/mL) compared to the untreated control. After Holm correction, significant reductions were confirmed for CXCL10/IP10 (*p* = 2.22 × 10^−9^), IL1β (*p* = 3.64 × 10^−6^), TNFα (*p* = 0.000445), and PAI-1 (*p* ≤ 4.83 × 10^−6^), whereas the reduction in IL6 did not remain significant.

Exposure to irradiation at single doses of 2 Gy or 8 Gy resulted in a reduction in the expression of all investigated proteins (IL6: 121.6 ± 0.5 pg/mL and 79.6 ± 12.1 pg/mL; CXCL10/IP10: 259.9 ± 32.9 pg/mL and 153.2 ± 18.9 pg/mL; IL1β: 109.3 ± 8.9 pg/mL and 104.2 ± 9.9 pg/mL; TNFα: 294.2 ± 0.1 pg/mL and 167.2 ± 63.6 pg/mL; and PAI-1: 5.2 ± 0.4 ng/mL and 4.3 ± 0.2 ng/mL for 2 and 8 Gy, respectively).

Combination treatment with sitagliptin and irradiation at 2 Gy resulted in lower expression levels of IL6 (65.7 ± 8.8 pg/mL), CXCL10/IP10 (101.4 ± 4.1 pg/mL), IL1β (52.2 ± 2.7 pg/mL), TNFα (184.9 ± 0.6 pg/mL), and PAI-1 (0.8 ± 0.02 ng/mL) compared to the cells after irradiation alone. Following the combination of sitagliptin and irradiation at a dose of 8 Gy, lower expression levels were detected for IL6 (55.0 ± 3.2 pg/mL), CXCL10/IP10 (78.7 ± 10.1 pg/mL), IL1β (65.7 ± 1.1 pg/mL), TNFα (64.1 ± 1.1 pg/mL), and PAI-1 (1.2 ± 0.04 ng/mL) compared to the cells irradiated at the same dose. Combination with sitagliptin resulted in a further reduction of several proteins compared to irradiation alone, although differences in IL6 and TNFα did not remain significant after Holm correction.

In untreated MDA-MB-231-INV cells, the constitutive levels of IL6 (53.6 ± 2.8 pg/mL), CXCL10/IP10 (59.4 ± 3.9 pg/mL), IL1β (28.7 ± 4.6 pg/mL), and TNFα (123.5 ± 3.8 pg/mL) were lower than in their parental counterparts. PAI-1 was not detected in the MDA-MB-231-INV cells. Treatment with sitagliptin alone did not induce any changes in the expression of any of the investigated proteins compared to the untreated control. In contrast, irradiation at a dose of 2 Gy upregulated the expression of IL6 (192.2 ± 34.4 pg/mL vs. 53.6 ± 2.8 pg/mL in the untreated control). However, this difference did not remain significant after Holm correction. Combination treatment with sitagliptin and irradiation at a dose of 2 Gy led to an increase in the expression of CXCL10/IP10 (75.5 ± 3.2 pg/mL) compared to irradiation alone (2 Gy) (32.2 ± 6.6 pg/mL). The significant upregulation of IL1β expression was detected after cell treatment with a combination of sitagliptin and irradiation at a dose of 2 Gy (66.3 ± 2.6 pg/mL) compared to cells exposed to irradiation (2 Gy) alone (15.8 ± 4.6 pg/mL) (Holm-adjusted *p* = 0.00176). The combination treatment with sitagliptin and 8 Gy of irradiation led to significantly increased expression levels of CXCL10/IP10 (124.3 ± 8.9 pg/mL) compared to the cells treated with irradiation (8 Gy) alone (31.4 ± 4.1 pg/mL) (Holm-adjusted *p* = 0.000830).

Consistent with these cell line- and treatment-dependent responses, three-way ANOVA identified significant interactions for CXCL10/IP10, IL1β, PAI-1, and TNFα, whereas no significant effects were detected for IL6. Neither untreated nor treated parental T47D or T47D-INV cells exhibited detectable levels of any of the investigated SASP-associated proteins.

### 3.4. Sitagliptin, Ionizing Radiation, and Autophagy Activation

**Figure 5:**
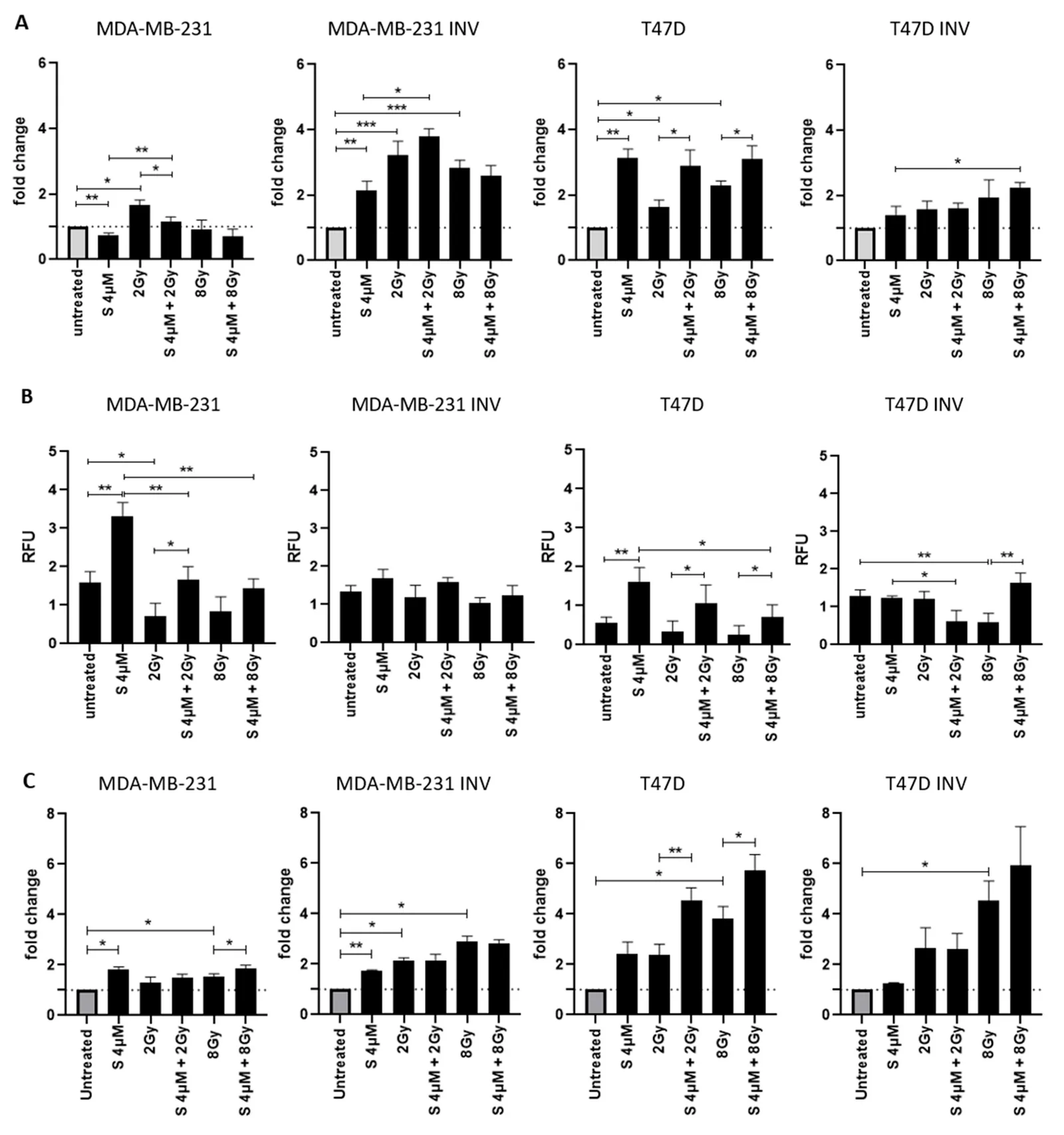
Autophagy development in BC cells after treatment with sitagliptin, irradiation, or both. (**A**) Microtubule-associated protein 1 light chain 3 alpha (LC3) gene expression was detected using the iQ™5 Real-Time PCR Detection System, 72 h after treatment with sitagliptin (4 µM) or irradiation (2 or 8 Gy) alone or both. ΔΔCt-values were also calculated. The bars are presented as the mean ± standard deviation of the fold change vs. the untreated control normalized to the housekeeping gene importin 8 (IPO8) after three independent experiments containing two technical replicates (*n* = 6). The gray bars show the untreated control, and the black bars represent the treated cells. (**B**) Autophagosomes were stained in BC cells treated as described in Section 2.9 of the Materials and Methods. The bars are presented as the mean ± standard deviation of the RFU after three independent experiments containing two technical replicates (*n* = 6). (**C**) Autophagosomes and autolysosomes were stained with the Autophagy Red Detection Kit and analyzed by FACS, as described in Section 2.9 of the Materials and Methods. The bars are presented as the mean ± standard deviation of the fold change vs. the untreated control, after two independent experiments containing two technical replicates (*n* = 4) (* *p* < 0.05; ** *p* < 0.01; *** *p* < 0.001). A dashed line at a fold change of 1 indicates the level of the untreated control to facilitate visualization of differences relative to the untreated control.

We next investigated autophagy-associated responses after cell treatment with sitagliptin, ionizing radiation, or both by analyzing MAP1LC3A (LC3) gene expression (Figure 5A). Sitagliptin alone caused a slight downregulation of MAP1LC3A (LC3) gene expression in parental MDA-MB-231 cells (0.75 ± 0.06-fold compared to the untreated control), whereas MDA-MB-231-INV and parental T47D cells possessed higher MAP1LC3A (LC3) expression levels (2.15 ± 0.2-fold for MDA-MB-231-INV and 3.14 ± 0.2-fold for parental T47D cells). T47D-INV cells did not show significant changes in their microtubule-associated protein 1 light chain 3 alpha (LC3) gene expression levels after treatment with sitagliptin (Figure 5A). In the MDA-MB-231 model, ANOVA confirmed significant effects of cell line and radiation dose, as well as significant cell line × sitagliptin, cell line × radiation dose, sitagliptin × radiation dose, and three-way interactions. Holm-adjusted comparisons revealed significantly higher MAP1LC3A expression in MDA-MB-231-INV cells compared to parental cells under all treatment conditions.

Radiation-dependent gene upregulations were determined in the parental MDA-MB-231 and T47D cells and MDA-MB-231-INV cells. Thus, cell exposure to ionizing radiation at 2 Gy caused the upregulation of the MAP1LC3A (LC3) gene in the mentioned cells (1.67 ± 0.11-fold for parental MDA-MB-231 cells, 3.23 ± 0.46-fold for MDA-MB-231-INV cells, and 1.63 ± 0.15-fold for parental T47D cells). T47D-INV cells did not show significant changes compared to the untreated control. Cell irradiation at a dose of 8 Gy resulted in increased expression levels of MAP1LC3A (LC3) in invasive MDA-MB-231-INV (2.85 ± 0.16-fold change) and parental T47D cells (2.3 ± 0.34-fold change), whereas parental MDA-MB-231 and invasive T47D cells had very similar gene expression levels to the untreated controls. In parental T47D cells, ANOVA confirmed significant effects of cell line, sitagliptin, and radiation dose, together with a significant cell line × sitagliptin interaction.

The combination treatment of the parental MDA-MB-231 cells with sitagliptin and ionizing radiation at 2 Gy attenuated the gene expression level compared to irradiation alone (1.16 ± 0.11-fold change for combination treatment vs. 1.67 ± 0.11-fold change for irradiation alone), whereas it was higher than in the cells treated with sitagliptin alone (0.75 ± 0.06-fold change). In contrast, in MDA-MB-231-INV cells, the MAP1LC3A (LC3) gene was upregulated in response to combination treatment (3.79 ± 0.17-fold change) compared to sitagliptin (2.15 ± 0.2-fold change) but not irradiation (2 Gy) alone (3.23 ± 0.46-fold change). The combined treatment with sitagliptin and irradiation at a higher dose of 8 Gy did not change MAP1LC3A (LC3) gene expression in parental MDA-MB-231 and metastatically active MDA-MB-231-INV and T47D-INV cells when compared to irradiation (8 Gy) alone. Holm-adjusted comparisons further showed that MAP1LC3A expression was significantly higher in parental T47D cells compared to T47D-INV cells following sitagliptin alone (*p* = 0.00203) and sitagliptin combined with 2 Gy (*p* = 0.0226). Parental T47D cells exhibited a significant ~1.4-fold upregulation of gene expression after the combination of sitagliptin and irradiation at a dose of 8 Gy compared to the cells that were only treated with irradiation at the same dose (Holm-adjusted *p* = 0.0237). However, there was no significant difference in gene expression in the cells exposed to sitagliptin alone or combination treatment. No corresponding effect was observed in T47D-INV cells.

To further assess the susceptibility of the cells to autophagosome formation induced by treatment with sitagliptin, ionizing radiation, or both (Figure 5B), we stained for autophagosomes. Parental MDA-MB-231 and T47D cells demonstrated different constitutive levels of autophagy-associated signal, whereas invasive BC cells showed comparable levels in untreated cells. Treatment with sitagliptin alone significantly increased the autophagy-associated signal in parental MDA-MB-231 (2.4 ± 0.26-fold change) and T47D cells (1.67 ± 0.03-fold change) compared to their untreated controls. MDA-MB-231-INV and T47D-INV cells demonstrated no changes after their exposure to sitagliptin alone. Consistently, ANOVA revealed significant cell line × sitagliptin interactions for both parental MDA-MB-231 versus MDA-MB-231-INV cells and parental T47D versus T47D-INV cells. Following sitagliptin treatment, the autophagy-associated signal was significantly higher in parental MDA-MB-231 cells compared to MDA-MB-231-INV cells (Holm-adjusted *p* = 2.28 × 10^−7^).

Irradiation at a dose of 2 Gy reduced the autophagy-associated signal in the parental MDA-MB-231 cells only (0.52 ± 0.03-fold change compared to the untreated control). All other investigated cells demonstrated no differences in the autophagy-associated signal after their treatment with ionizing radiation, independent of the dose.

Parental MDA-MB-231 and T47D cells showed higher autophagy-associated signals after combination treatment with sitagliptin and irradiation (2 Gy) compared to irradiation alone (~2.5- and ~1.9-fold, respectively). However, the autophagy-associated signals after combination treatment were ~1.9- and ~1.4-fold lower in parental MDA-MB-231 and T47D cells, respectively, in comparison with the cells treated with sitagliptin alone. Neither MDA-MB-231-INV nor T47D-INV demonstrated any changes in the autophagy-associated signal compared to irradiation alone. The combination of sitagliptin and a higher dose of irradiation at 8 Gy did not affect the autophagy-associated signal in parental and invasive MDA-MB-231 cells compared to irradiation alone. In contrast, parental T47D and T47D-INV cells showed a significant upregulation of the autophagy-associated signal (~1.6- and ~1.8-fold, respectively) upon combination treatment with sitagliptin and irradiation at 8 Gy. ANOVA supported these treatment- and cell line-dependent responses, including significant interaction effects in both models. Holm-adjusted comparisons further identified significant differences between parental and invasive T47D cells following 2 Gy (*p* = 0.0186) and following sitagliptin combined with 8 Gy (*p* = 0.0261).

Additional staining was performed to assess the formation of autophagosomes and autolysosomes, followed by flow cytometry, and the corresponding results are presented in Figure 5C.

Parental MDA-MB-231 cells exhibited only limited changes in the autophagy-associated signal across all treatment conditions. Treatment with sitagliptin alone (~0.82-fold upregulation) and irradiation at a single dose of 8 Gy (~0.37-fold upregulation) resulted in an increase in the autophagy-associated signal compared to the untreated control. Furthermore, combined treatment with sitagliptin and irradiation at 8 Gy induced a ~0.49-fold increase in the autophagy-associated signal compared to the cells treated with irradiation (8 Gy) alone. ANOVA confirmed significant effects of cell line, sitagliptin, and radiation dose, together with significant cell line × radiation dose and sitagliptin × radiation dose interactions.

Similarly, MDA-MB-231-INV cells displayed a slight increase in the autophagy-associated signal following treatment with sitagliptin alone (~0.76-fold upregulation) as well as after irradiation at 2 Gy (~1.17-fold upregulation) or 8 Gy (~1.91-fold upregulation) compared to the untreated control. No significant changes were observed after combination treatment using any doses of irradiation in comparison with irradiation alone. Holm-adjusted comparisons revealed significantly higher autophagy-associated signals in MDA-MB-231-INV cells compared to parental cells following 2 Gy (*p* = 0.00187), sitagliptin and 2 Gy (*p* = 0.0134), 8 Gy (*p* = 2.40 × 10^−5^), and sitagliptin and 8 Gy (*p* = 8.03 × 10^−4^).

In contrast, consistent with the findings shown in Figure 5A,B, parental T47D cells showed a dose-dependent increase in the autophagy-associated signal after irradiation (~1.39- and ~2.81-fold upregulation for 2 and 8 Gy, respectively) compared to the untreated control. The combination of sitagliptin and irradiation at 2 Gy resulted in an additional ~2.2-fold increase in the autophagy-associated signal compared to cells treated at 2 Gy alone. The combined treatment with sitagliptin and 8 Gy led to a ~1.93-fold increase in the autophagy-associated signal compared to the cells exposed to irradiation at 8 Gy alone.

T47D-INV cells demonstrated a dose-dependent increase in the autophagy-associated signal, with ~1.67-fold and ~3.54-fold upregulation followed by cell exposure to ionizing radiation at 2 or 8 Gy compared to the untreated control. However, the combination of sitagliptin with irradiation at either 2 or 8 Gy did not result in any significant changes in the autophagy-associated signal compared to the respective irradiation-only groups. ANOVA confirmed significant overall effects of sitagliptin and radiation dose and a significant cell line × sitagliptin interaction in the parental and invasive T47D cells. However, individual comparisons between treatments with and without sitagliptin at the respective radiation doses, as well as comparisons between parental and INV cells, did not remain significant after Holm correction.

## 4. Discussion

Both T2DM and breast cancer represent the most common diseases in the female population worldwide [29,30]. Epidemiological studies have clearly demonstrated that T2DM is associated with an increased risk of cancer development [29], but this metabolic disorder also negatively influences clinical outcomes in cancer patients. Moreover, a meta-analysis of the multitude of studies showed that pre-existing T2DM had an impact on all-cause mortality in BC patients (pooled hazard ratio [HR], 1.49; 95% CI, 1.35 to 1.65) compared with their nondiabetic counterparts [31]. It was assumed that this could be associated with T2DM-related comorbidities [32], but we also hypothesize that T2DM itself and antidiabetic drugs can change the efficacy of anticancer therapeutic approaches, such as chemotherapy, radiotherapy, and hormonal therapy. Novel antidiabetic medications have been developed according to the molecular mechanisms regulating glucose levels in patients. However, these mechanisms can also be implicated in the processes of carcinogenesis, tumor progression, and therapy responses [33,34,35].

As a transmembrane glycoprotein, DPP4/CD26 possesses enzymatic activities resulting in the degradation of incretin hormones involved in the regulation of blood glucose levels [13,36,37]. Therefore, the use of DPP4/CD26 inhibition was proposed for the treatment of T2DM. Besides incretins, the protease DPP4/CD26 may be involved in the regulation of other hormones, growth factors, and chemokines associated with cancer progression. Therefore, it might be possible that DPP4 inhibitors have undesirable effects in cancer patients receiving antitumor therapy. To achieve specific DPP4/CD26 inhibition, the humanized antibody to CD26 (YS110) was developed and used in patients with malignant mesothelioma [19,38]. A phase I clinical trial demonstrated good drug tolerance and disease stabilization. Therefore, it was assumed that DPP4/CD26 inhibitors would not lead to the progression of malignant tumors. However, further investigations of the role of DPP4/CD26 molecules in cancer biology showed highly controversial results. Some studies reported that the overexpression of DPP4/CD26 was associated with the inhibition of tumor growth [39,40], whereas others revealed a link between DPP4/CD26 overexpression and tumor progression [16,17,41,42]. In parallel, it was also shown that DPP4/CD26 deficiency or its treatment-dependent inhibition promoted cancer cell survival and is associated with poor prognosis in BC patients [18,43]. Since DPP4/CD26 is implicated in the processes of invasiveness and metastasis formation, its expression was evaluated in breast carcinoma cells with enhanced metastatic capacities [21]. Our findings demonstrate the upregulation of DPP4/CD26 in INV cells compared to their parental counterparts, which correlates with other reports on the role of DPP4/CD26 in cancer cell stemness, epithelial-to-mesenchymal transition, and metastatic spread in malignant tumors [17,44,45,46]. The additional proteome array analysis, with the results presented in the Supplementary Materials, also indicated increased DPP4 levels in INV cells, particularly in the cell culture supernatant of T47D INV cells compared to their parental counterparts. Interestingly, the increase in DPP4 was more pronounced in the T47D cell culture supernatant than in the corresponding cell lysates. Although these descriptive data require confirmation in independent experiments, they suggest that differences in DPP4/CD26 associated with the invasive phenotype may not be restricted to cellular protein levels but could also involve the soluble form of DPP4/CD26 released into the TME.

Our previously published study unveiled the appearance of stemness capacities in INV cells accompanied by augmented invasiveness and abilities to metastasize [21]. Taking these data into account, it is logical to assume that the inhibition of DPP4/CD26 can affect the survival abilities of metastatic carcinoma cells. Unfortunately, conflicting results were obtained in preclinical and clinical studies when DPP4 inhibitors were used [16,17,18,30,42,47]. It is interesting to note that patients with malignant tumors and high DPP4/CD26 expression levels demonstrated better survival in the groups that received DPP4 inhibitors [19]. However, the effects of the use of these inhibitors in breast cancer patients, particularly those with triple-negative tumors, are still unclear and under investigation. Since BC is a highly heterogeneous malignancy, responses to DPP4 inhibitors in tumors belonging to different BC subtypes can vary markedly.

Furthermore, it is still undefined as to how DPP-4is change therapy responses when they are combined with conventional antitumor approaches. Although there are few scientific reports on the efficacy of the combination of DPP-4is and chemotherapeutics, the majority of the findings are controversial, conflicting, and therefore inconclusive [48,49,50,51,52]. To our knowledge, there is only one publication on the role of DPP-4i vildagliptin in the sensitization of murine tumors (melanoma, Lewis lung, and breast carcinomas) to ionizing radiation via the promotion of T-cell infiltration [47]. Unfortunately, there are no reports or studies on the direct influence of DPP4 inhibitors on the radiation response of carcinoma cells.

Recently, Wang et al. [53] found that DPP-4is saxagliptin and sitagliptin activated the NRF2-associated antioxidant response in carcinoma cells, resulting in enhanced metastasis in animal models. Since NRF2-related molecular pathways can protect cells from the cytotoxic and anti-proliferative activities of ionizing radiation [54,55,56,57,58,59], we hypothesize that the DPP4 inhibitor sitagliptin can affect the radiation responses of carcinoma cells. However, NRF2 signaling was not investigated in the present study, and its potential contribution to the observed effects of sitagliptin on the radiation response therefore remains to be elucidated in future mechanistic studies.

Our study shows that sitagliptin in combination with irradiation caused different cell responses in triple-negative and luminal A breast carcinoma cells, with enhanced or reduced responses to irradiation observed depending on the specific treatment conditions. Although cell proliferation and short-term cell viability were not markedly affected by sitagliptin, treatment-associated increases in long-term reproductive clonogenic survival were observed in triple-negative BC cells under specific conditions. In contrast, neither parental nor invasive luminal A BC cells showed enhanced cell survival after sitagliptin treatment. Interestingly, increased clonogenic survival was observed in triple-negative BC cells when sitagliptin was combined with ionizing radiation at the lower dose, whereas in luminal A cells, these effects were not observed. As recently shown, triple-negative MDA-MB-231 cells possess a mesenchymal phenotype that is enhanced in INV cells, and luminal A T47D cells have an epithelial phenotype that transitions to the hybrid phenotype in INV cells [21,60]. Therefore, our findings suggest that the influence of sitagliptin on the radiation response may depend on the phenotypic state of BC cells, particularly in cells displaying a mesenchymal phenotype. Comparable effects were not observed in BC cells with epithelial or hybrid phenotypes.

Since the investigated BC cells demonstrated controversial treatment responses after the administration of sitagliptin, irradiation, or both, we decided to evaluate two mechanisms (senescence and autophagy) that can be implicated in both cell death and survival after cell exposure to cytotoxic agents [61,62,63]. It was found that both sitagliptin and irradiation are contributors to senescence development in parental MDA-MB-231, whereas other investigated cells were not subjected to senescent reprogramming. Although senescence is considered an alternative cellular process allowing cells to evade apoptotic cell death, carcinoma cells are inhibited in their proliferation [63,64]. In recent years, the senescence-associated secretory phenotype (SASP) has emerged as an important focus in TNBC, as many standard therapies are accompanied by therapy-induced senescence (TIS) rather than apoptosis. Although senescent cells undergo permanent cell cycle arrest, they remain metabolically active and secrete a broad range of SASP factors that can profoundly remodel the tumor microenvironment (TME) [65,66]. Importantly, SASP has been shown to exert a dual role in TNBC. While therapy-induced senescence initially suppresses tumor cell proliferation, sustained SASP secretion can promote a pro-tumorigenic microenvironment, enhance therapy resistance, and increase metastatic potential [67]. This has made SASP modulation a promising area of translational research in TNBC. The broader proteome array profiles did not consistently correspond to the changes observed for the selected SASP-associated proteins or other senescence-associated parameters. However, the proteome arrays characterized basal differences between parental and INV cells and assessed a broader set of proteins in different sample compartments. Therefore, direct correspondence with the treatment-dependent SASP analysis would not necessarily be expected. Moreover, as the arrays were performed as a single exploratory experiment, these findings should be interpreted descriptively.

Indeed, senescence development in the parental MDA-MB-231 cells in response to treatment with irradiation and its combination with sitagliptin was accompanied by a significant reduction in cell viability compared to the untreated control or sitagliptin alone. Despite this initial growth inhibition, the surviving cell population retained the ability to form colonies, suggesting that the treatment-induced senescence-associated response was not accompanied by a complete loss of long-term reproductive capacity. This observation was supported by a marked reduction in the expression of key SASP factors, such as IL-6, CXCL10, IL-1β, TNFα, and PAI-1, after sitagliptin treatment alone and in combination with irradiation at 2 or 8 Gy. Because senescence is typically associated with the sustained secretion of SASP factors that help maintain stable cell cycle arrest, the reduced levels of several of these mediators suggest that sitagliptin may modify the senescence-associated response and potentially facilitate recovery from treatment-induced growth arrest. Interestingly, although sitagliptin worsened the cytotoxic effects of low-dose irradiation, the surviving cells exhibited enhanced clonogenic capacity. This indicates that they regained long-term proliferative potential. Together, these findings suggest that the senescence-associated response may not necessarily result in an irreversible loss of proliferative capacity in the surviving cell population. This state may provide radioprotection to the surviving tumor cell population by facilitating recovery from irradiation-induced growth arrest. This recovery enables long-term colony formation. Hence, we hypothesized that the restoration of the replication capacities of the cells may be due to autophagy activation in the treated cells. It is currently known that autophagy is upregulated in cells to remove damage that occurred after treatment, helping the cells to survive [68]. Both senescence and autophagy can either lead to cell death or protect cells from cytotoxic agents. It was first suggested that the regulation of autophagy could improve the therapy responses of carcinoma cells [69,70,71,72,73], but later, several publications showed that the inhibition of autophagy does not necessarily lead to the sensitization of cells to ionizing radiation or chemotherapeutic agents and plays a non-protective role [63,73,74,75,76]. Parental MDA-MB-231 cells exhibited both senescence- and autophagy-associated responses following treatment with sitagliptin alone or in combination with ionizing radiation, while surviving cells retained long-term clonogenic capacity. In line with these findings, sitagliptin increased autophagic activity in parental T47D cells both as a single treatment and in combination with irradiation. In contrast, the corresponding INV cells showed less pronounced autophagy-associated responses to sitagliptin alone, suggesting that acquisition of an invasive phenotype may be associated with an altered capacity to activate autophagy in response to sitagliptin. Accordingly, BC cells with invasive capacities were generally not characterized by increased susceptibility to senescence and/or autophagy, highlighting the context-dependent nature of the cellular response to sitagliptin. These paradoxical observations described in the literature and observed in our own experiments allow us to conclude that both treatment-caused senescence and autophagy can vary depending on cell type and the kind of treatment. Other types of intracellular molecular signaling can contribute to the functional abilities of senescence and autophagy, resulting in either cell death or protection from cytotoxic agents, and these mechanisms should carefully be elucidated.

Summarizing the obtained data, we conclude that BC cells with enhanced invasive and migratory capacities are characterized by upregulation of DPP4/CD26 expression. Our findings indicate that DPP4/CD26 inhibition with sitagliptin may be associated with differential responses to irradiation, with an improved response to irradiation in luminal A BC cells and an attenuated response in TNBC cells under specific treatment conditions. Overall, these different effects of sitagliptin did not consistently correlate with the extent of senescence and/or autophagy observed after treatment, suggesting that additional molecular mechanisms may contribute to these responses.

## Data Availability

The raw data supporting the conclusions of this article will be made available by the authors upon request. The data are not publicly available due to privacy constraints, including data protection requirements associated with the project. Requests for data access will be evaluated on a case-by-case basis and subjected to appropriate approvals.

## Supplementary Materials

The following supporting information can be downloaded at: https://www.mdpi.com/article/10.3390/cells15201879/s1, Figure S1: Gating strategy for CD26; Figure S2: Gating strategy using Autophagy Red Assay; Additional experiment: Proteome array; Figure S3: Proteome array.
