# Supplementary material for: The Impact of the Antidiabetic Agent Sitagliptin on the Radiation Response of Breast Carcinoma Cells with Different Metastatic Capacities
Source: Cells. 2026 Oct 15;15(20):1879. doi: 10.3390/cells15201879 (PMC13702927; doi:10.3390/cells15201879)
Supplement: Supplementary file 1 [file cells-15-01879-s001.zip › cells-4554036-supplementary.pdf]

Supplemental material

Figure S1

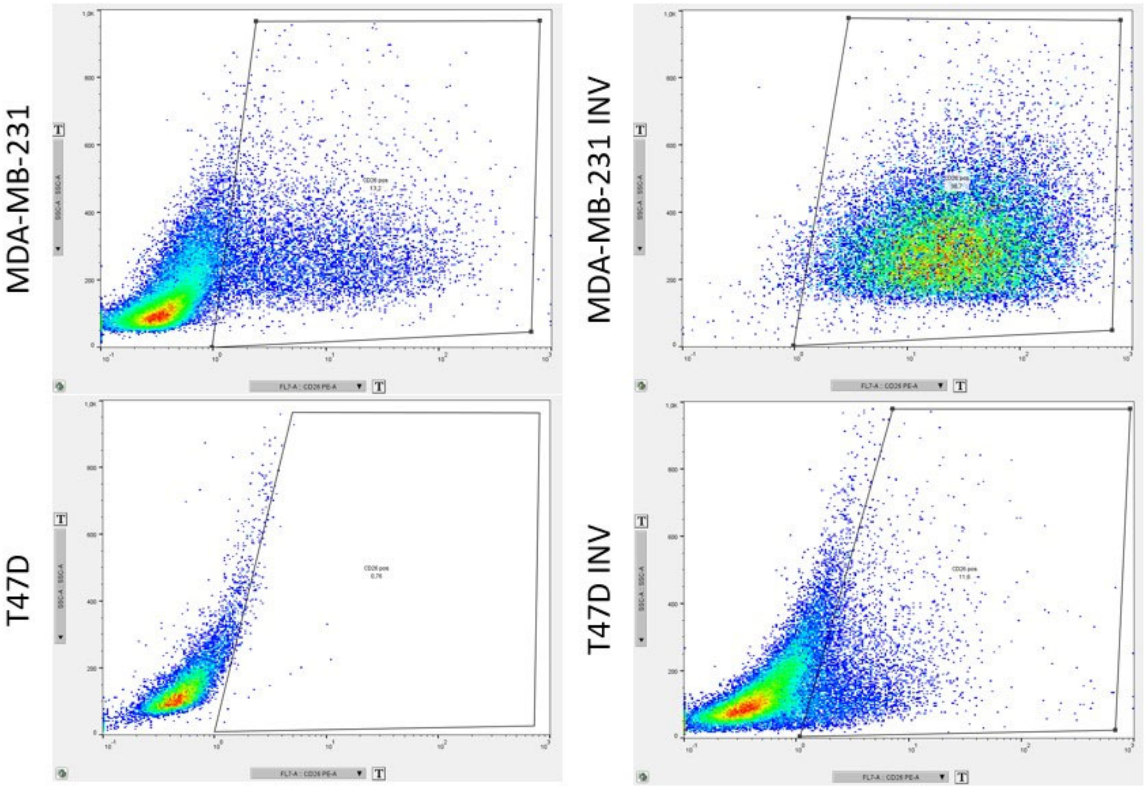

Figure S1. Gating strategy for CD26. FlowJo software was used to gate CD26/PE-positive cells. The mean fluorescence intensity (MFI) was also calculated in FlowJo and added as a second figure.

Figure S2

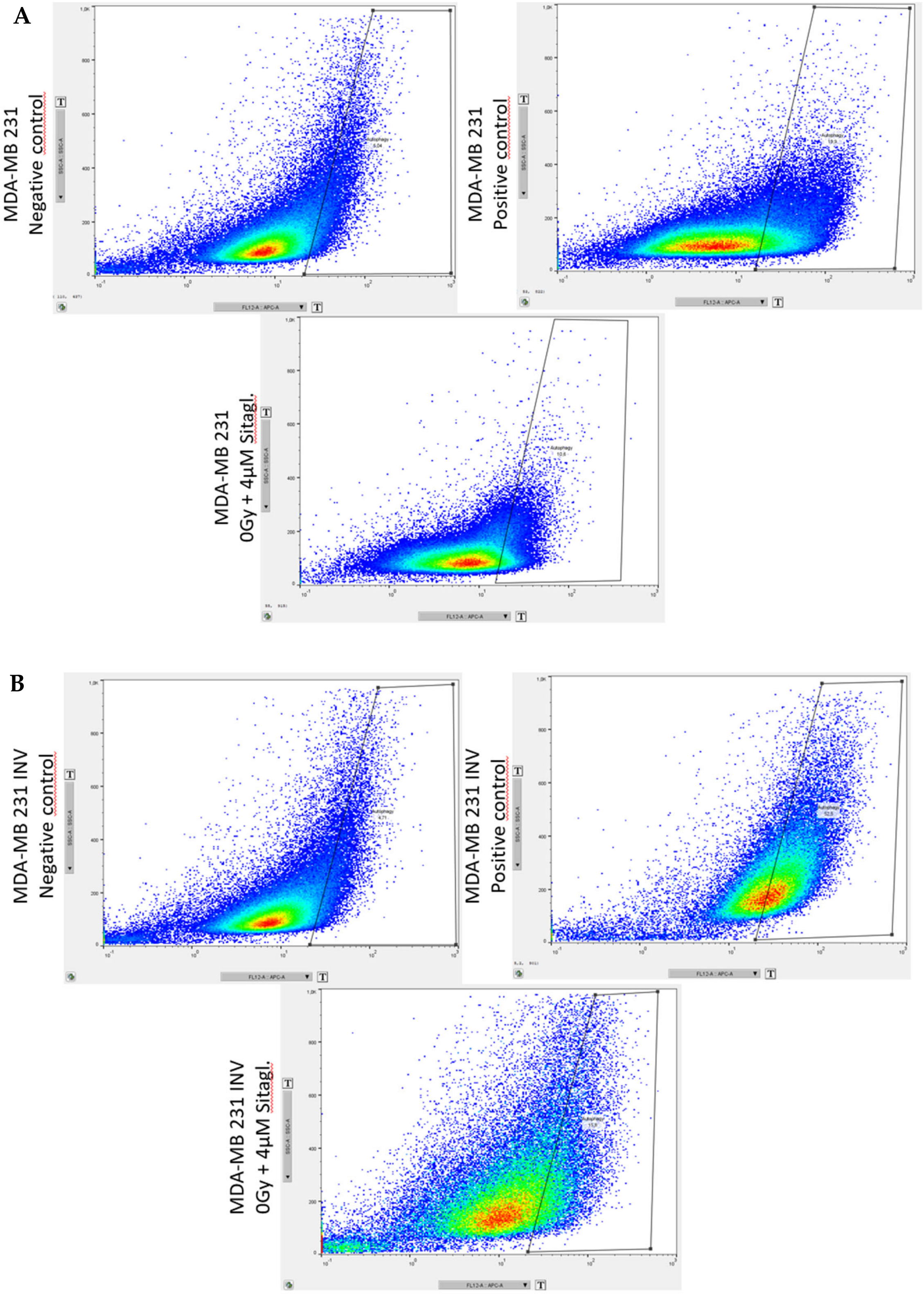
A
MDA-MB 231
Negative control
MDA-MB 231
Positive control
MDA-MB 231
0Gy + 4µM Sitagl.
B
MDA-MB 231 INV
Negative control
MDA-MB 231 INV
Positive control
MDA-MB 231 INV
0Gy + 4µM Sitagl.

C

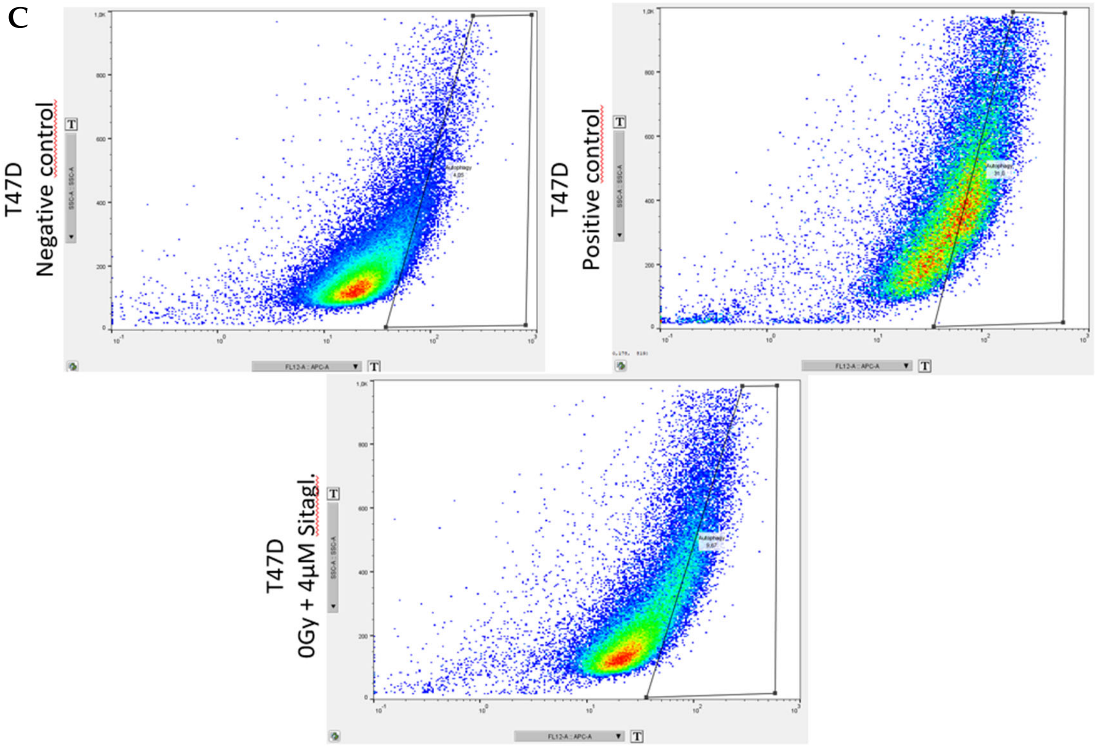

D

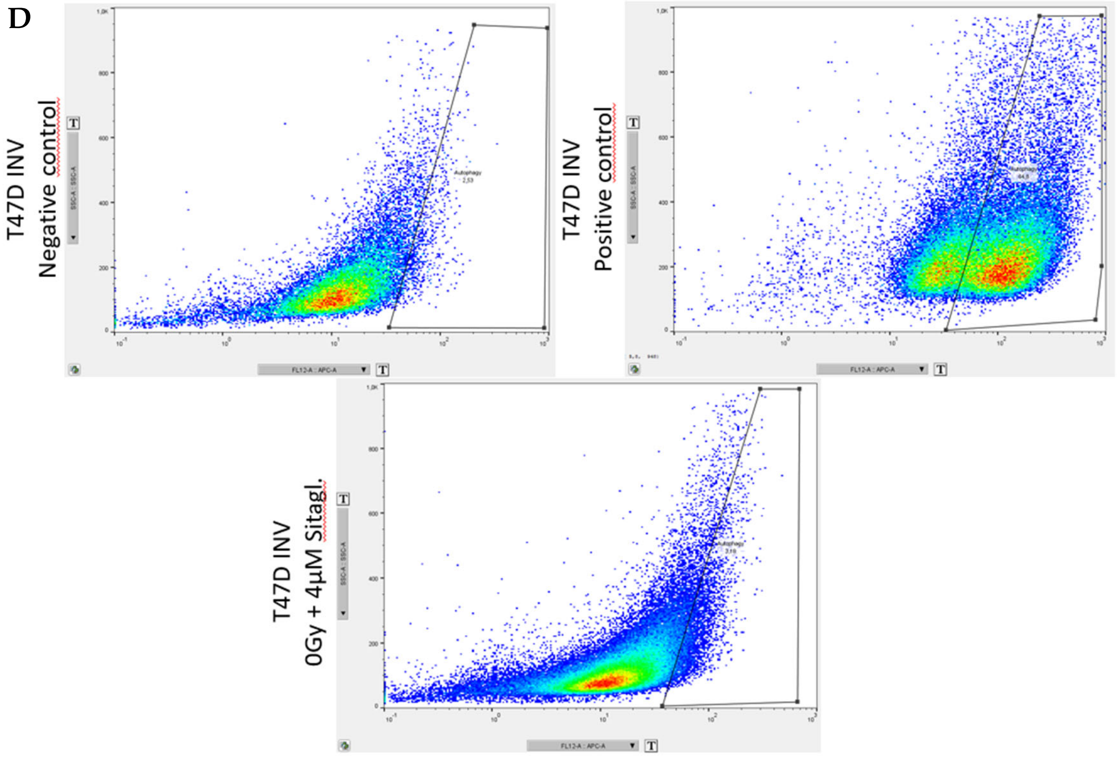

Figure S2. Gating strategy using Autophagy Red Assay. FlowJo software was used to gate autophagic cells. APC-positive cells were gated relative to negative (0.01% DMSO) and positive (Rapamycin (500nM) and Chloroquine (60µM)) controls from all cells. For each cell line, gating with respect to positive and negative controls was used. One example is shown for each. Gating for (A) MDA-MB 231, (B) MDA-MB 231 INV, (C) T47D, and (D) T47D INV cells.

Additional experiment. Proteome array

Material and method:

To compare the protein profiles of parental and INV BC cells, proteome array analyses were performed using the Human XL Cytokine Array Kit and the Human Adipokine Array Kit (R&D Systems, Minneapolis, MN, USA). Parental MDA-MB-231 and T47D cells and their corresponding INV cells were plated under standard culture conditions. After 24 h, cell culture supernatants were analyzed using the Human XL Cytokine Array Kit, whereas cell lysates were analyzed using the Human Adipokine Array Kit according to the manufacturer's protocols.

Spot densities were analyzed using Image Studio™ Lite 5.0 (LI-COR Biotechnology, Lincoln, NE, USA). Following background correction, the integrated density value (IDV) was calculated as the ratio of normalized protein spot densities in INV cells relative to the corresponding parental cells. Each protein was represented by duplicate spots, indicated by red squares in the supplementary figures.

Result:

Protein profiles of parental and INV BC cells were analyzed using adipokine and cytokine arrays (Figure S3). Analysis of cell lysates showed differences in adipokine-associated protein levels between parental and INV cells (Figure S3 A and B). In MDA-MB-231-INV cells, TNF-α showed an approximately 6.5-fold increase compared to parental cells. Increases were also observed for Fetuin B, FGF basic, IL-1β, IL-10, Nidogen-1/Entactin, PBEF/Visfatin, Resistin, Serpin A8/AGT, Serpin E1/PAI-1, and TIMP-1. In T47D-INV cells, the largest increases were observed for IL-6 (~15.6-fold), Pentraxin-3/TSG-14 (~11.2-fold), DPP4 (~5.6-fold), PBEF/Visfatin (~4.8-fold), and Nidogen-1/Entactin (~4.4-fold).

Analysis of cell culture supernatants showed differences in secreted protein levels between parental and INV cells (Figure S3 C and D). In MDA-MB-231-INV cells, the largest increases compared to parental cells were observed for TNF-α (~20.8-fold), RANTES (~13.3-fold), MMP-9 (~8.0-fold), and DPP4 (~5.4-fold). Increased levels of CD14, Endoglin, ICAM-1, IL-32, MIP-3α, Relaxin-2, Resistin, and uPAR were also observed. In T47D-INV cells, the largest increases were observed for DPP4 (~10.9-fold) and CD14 (~4.4-fold), with smaller changes observed for the remaining proteins shown in Figure S3D.

The proteome arrays were performed as a single experiment with duplicate protein spots. Therefore, the results are presented descriptively without statistical analysis.

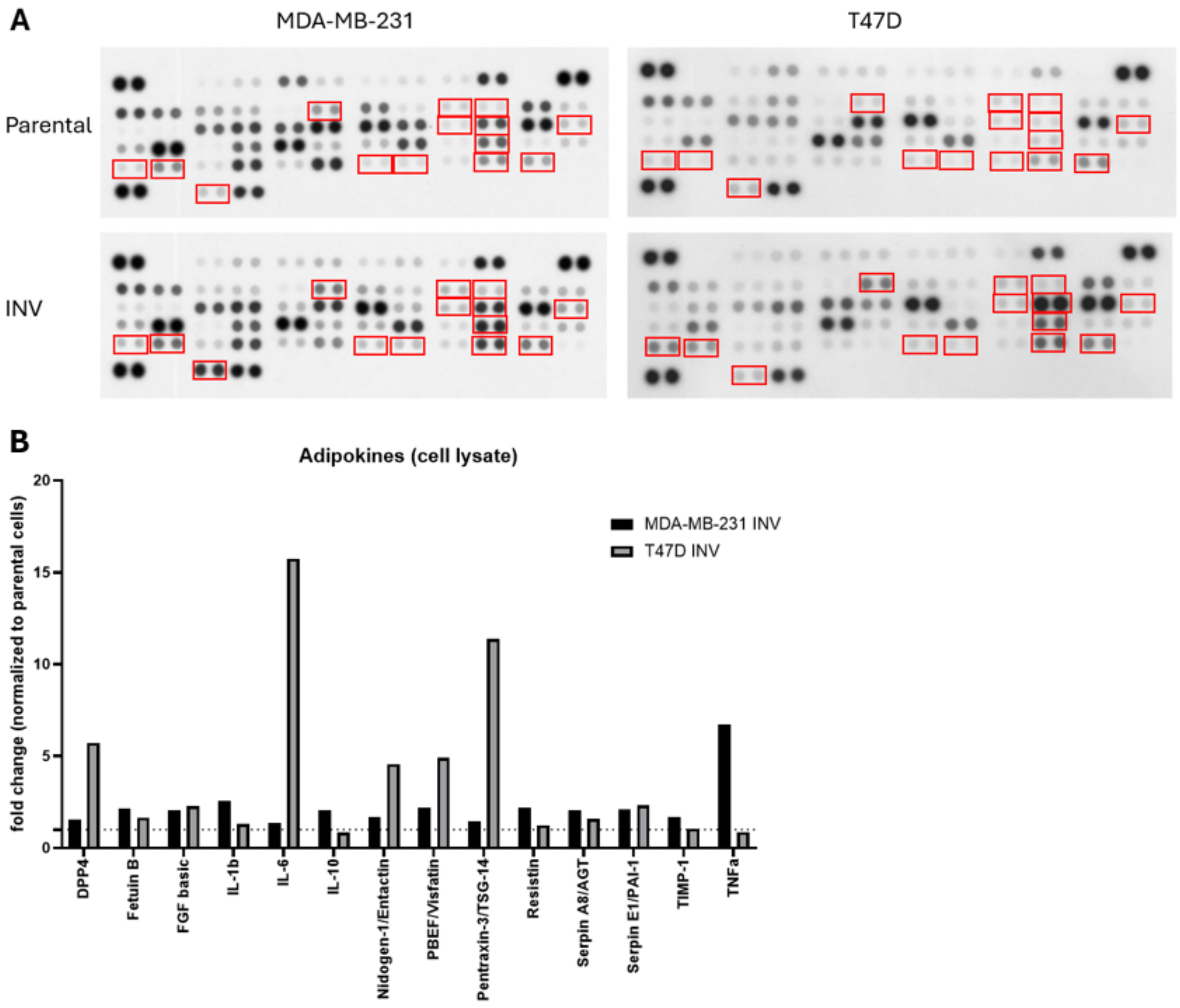
A
MDA-MB-231
T47D
Parental
INV
B
Adipokines (cell lysate)
fold change (normalized to parental cells)
20
15
10
5
0
MDA-MB-231 INV
T47D INV
DPP4
Fetuin B
FGF basic
IL-1b
IL-6
IL-10
Nidogen-1/Entactin
PBEF/Visfatin
Pentraxin-3/TSG-14
Resistin
Serpin A8/AGT
Serpin E1/PAI-1
TIMP-1
TNFa

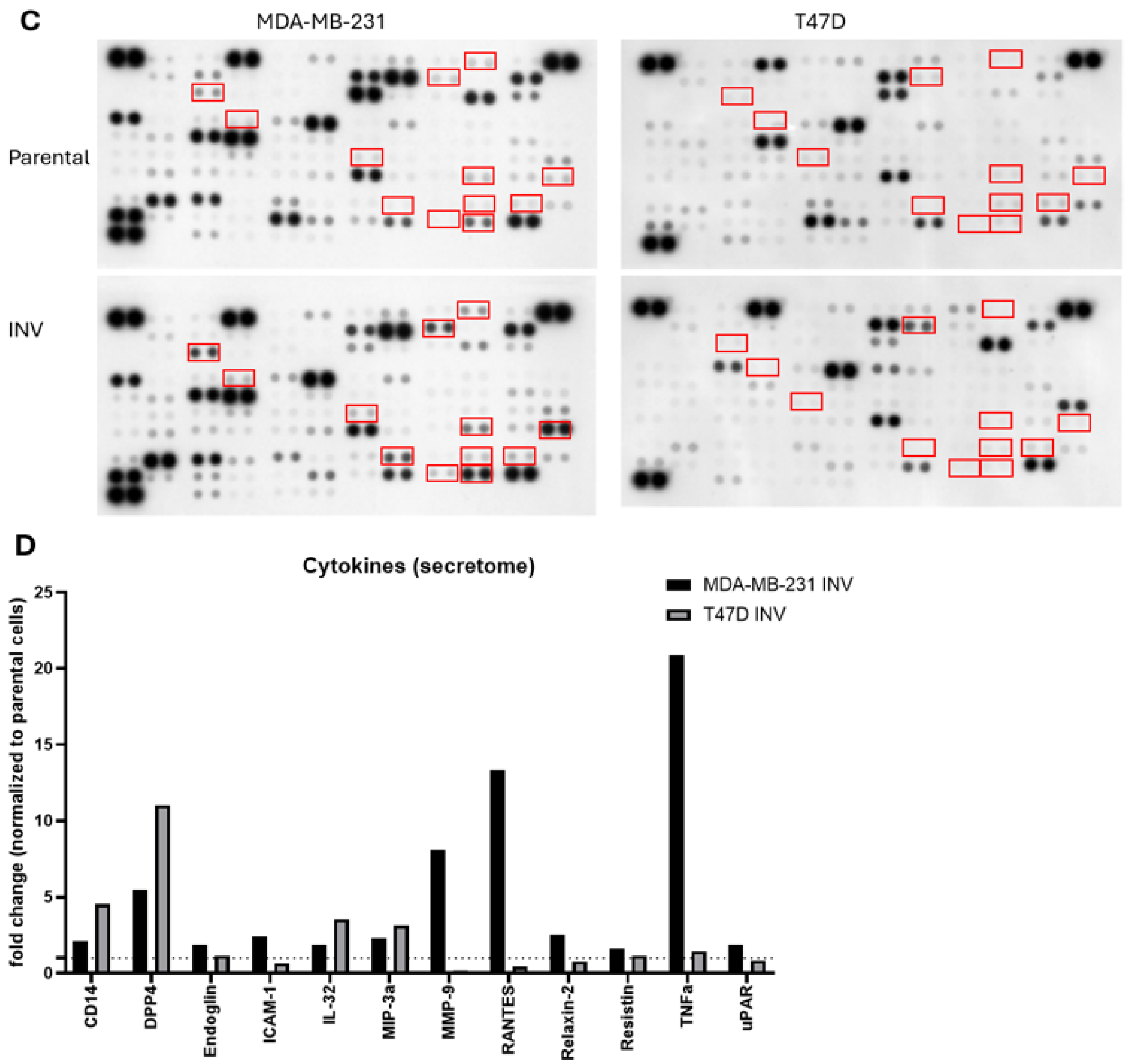

Figure S3. Proteome array. Parental and INV BC cells were plated and 24h later lysed (Human Adipokine Array Kit) or the supernatant was analyzed (Human XL Cytokine Array Kit) according to the manufacturer´s protocol. Spot densities were analyzed by the Image StudioTM Lite 5.0 (LI-COR Biotechnology, Lincoln, NB, USA). The Integrated Density Value (IDV) was obtained as a ratio of normalized protein spot densities in parental and INV cells after background correction. (A) Adipokines: Protein spots (in duplicates) are marked by red squares (B) Adipokines: Black bars represent the MDA-MB-231-INV cells. Gray bars represent the T47D-INV cells. The fold change to the respective parental cells was calculated. (C) Cytokines: Protein spots (in duplicates) are marked by red squares. (D) Cytokines: Black bars represent the MDA-MB-231-INV cells. Gray bars represent the T47D-INV cells. The fold change to the respective parental cells was calculated.
